# TRF1 and TRF2 use different mechanisms to find telomeric DNA but share a novel mechanism to search for protein partners at telomeres

**DOI:** 10.1093/nar/gkt1132

**Published:** 2013-11-22

**Authors:** Jiangguo Lin, Preston Countryman, Noah Buncher, Parminder Kaur, Longjiang E, Yiyun Zhang, Greg Gibson, Changjiang You, Simon C. Watkins, Jacob Piehler, Patricia L. Opresko, Neil M. Kad, Hong Wang

**Affiliations:** ^1^Physics Department, North Carolina State University, Raleigh, NC 27695, USA, ^2^Department of Environmental and Occupational Health, University of Pittsburgh Graduate School of Public Health, Pittsburgh, PA 15219, USA, ^3^Electric and Computer Engineering Department, University of North Carolina at Charlotte, Charlotte, NC 28223, USA, ^4^Department of Industrial and System Engineering, North Carolina State University, Raleigh, NC 27695, USA, ^5^Department of Cell Biology, Center for Biologic Imaging, University of Pittsburgh Graduate School of Public Health, Pittsburgh, PA 15219, USA, ^6^Division of Biophysics, Universität Osnabrück, Barbarstrasse 11, 49076, Osnabrück, Germany and ^7^School of Biological Sciences, University of Essex, Colchester, Essex CO4 3SQ UK

## Abstract

Human telomeres are maintained by the shelterin protein complex in which TRF1 and TRF2 bind directly to duplex telomeric DNA. How these proteins find telomeric sequences among a genome of billions of base pairs and how they find protein partners to form the shelterin complex remains uncertain. Using single-molecule fluorescence imaging of quantum dot-labeled TRF1 and TRF2, we study how these proteins locate TTAGGG repeats on DNA tightropes. By virtue of its basic domain TRF2 performs an extensive 1D search on nontelomeric DNA, whereas TRF1’s 1D search is limited. Unlike the stable and static associations observed for other proteins at specific binding sites, TRF proteins possess reduced binding stability marked by transient binding (∼9–17 s) and slow 1D diffusion on specific telomeric regions. These slow diffusion constants yield activation energy barriers to sliding ∼2.8–3.6 κ_B_T greater than those for nontelomeric DNA. We propose that the TRF proteins use 1D sliding to find protein partners and assemble the shelterin complex, which in turn stabilizes the interaction with specific telomeric DNA. This ‘tag-team proofreading’ represents a more general mechanism to ensure a specific set of proteins interact with each other on long repetitive specific DNA sequences without requiring external energy sources.

## INTRODUCTION

Telomeres play a crucial role in maintaining the stability of linear chromosomes (1,2). Loss of telomere function can activate DNA repair processes, leading to nucleolytic degradation of natural chromosome ends and their end-to-end fusion (3). Telomere dysfunction and associated chromosomal abnormalities have been strongly associated with age-related degenerative diseases and cancer (4,5). In a typical human somatic cell, the telomeric repeat sequence TTAGGG is ∼2–15 kb in length with a 3′-overhang of ∼100–200 nt (6). This 3′-overhang serves as a substrate for the reverse transcriptase telomerase, which replicates the telomeric sequence by using an internal RNA subunit as a template to direct the DNA synthesis (1,7–9). A specialized protein complex, shelterin (or telosome) binds to and protects the chromosome ends (2,10). The shelterin complex in humans consists of six core proteins: TRF1, TRF2, POT1, TIN2, TPP1 and RAP1 (1,11).

**Figure 1.**
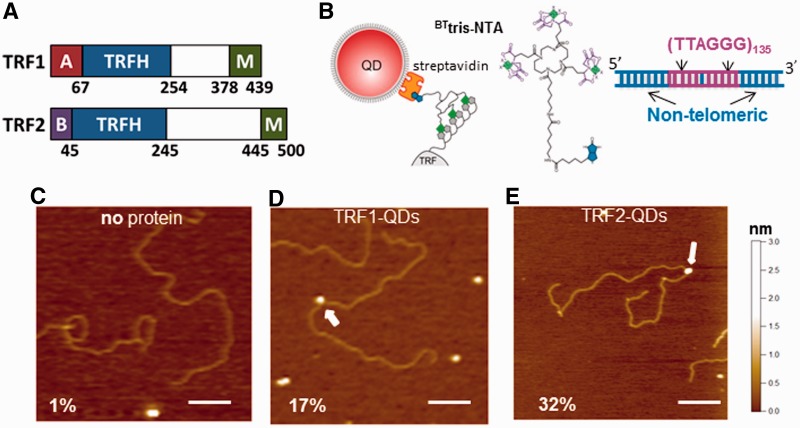
TRF1- and TRF2-QDs retain DNA-binding activity. (**A**) Schematic representations of the domain structures of TRF1 and TRF2. A: Acidic domain, B: Basic domain. M: Myb type domain. (**B**) Schematic representations of TRF1- and TRF2-QD conjugates (left), ^BT^tris-NTA compound (middle) and the DNA substrate (T270) with two tandem (TTAGGG)_135_ repeats connected by a short linker region (right, 5.4 kb in length). (C–E) Representative AFM images of DNA in the presence of (**C)** only QDs and ^BT^tris-NTA compound, (**D**) TRF1-QDs or (**E**) TRF2-QDs. The scale bar is 200 nm. White arrows point to QDs bound to DNA. The numbers in (C–E) indicate the percent of DNA molecules bound with QDs in each condition. The total numbers of complexes analyzed were 200, 250 and 250, for no protein, TRF1-QDs and TRF2-QDs, respectively.

TRF1 and TRF2 are the only proteins in the shelterin complex that make high-affinity contact with double-stranded telomeric DNA (12,13). TRF1 negatively regulates telomere length and promotes telomere replication (14). Whereas, TRF2 caps and protects chromosome ends (11), in addition to regulating telomere length (15). Removal of TRF2 from the telomeres results in loss of the 3′-overhang, covalent fusion of telomeres and induction of ATM and p53 dependent apoptosis (16,17). Both TRF1 and TRF2 contain a TRFH domain that mediates homodimerization and a Myb type domain that sequence-specifically binds to telomeric DNA (Figure 1A) (12). However, these two proteins differ at their N-termini, where TRF1 and TRF2 are rich in acidic and basic residues, respectively. Previous electron microscopy (EM) and atomic force microscopy (AFM) studies established that both TRF1 and TRF2 play important architectural roles at telomeres (18–21). TRF1 forms protein filaments on longer telomeric repeats (≥27 repeats) and promotes parallel pairing of telomeric tracts (19). *In vitro*, TRF2 can remodel linear telomeric DNA into T-loops (20).

A previous cell-based study of TRF1 and TRF2 using fluorescence recovery after photobleaching and fluorescence loss in photobleaching suggested that TRF1 and TRF2 interact with telomeres in a dynamic fashion (22). Although TRF1 and TRF2 are proposed to have extra-telomeric functions, they preferentially localize to the TTAGGG repeat sequences whether these target sites are at interstitial regions or at chromosome ends (23–25). Once telomeric sequences are located, TRF1 and TRF2 must find protein partners to form the shelterin complex and to regulate the functions of other DNA-binding proteins at telomeres (26–28). Despite recent advancements in the understanding of functions of TRF1 and TRF2, it is still unclear how TRF1 and TRF2 are able to find telomeric sequences and protein partners in a genome of billions of base pairs.

Accumulating evidence suggests that a protein can use one-dimensional (1D) sliding (correlated translocation while maintaining continuous DNA contact), jumping (noncorrelated detachment and reattachment) or hopping (correlated detachment and reattachment) to navigate through the vast excess of nonspecific DNA sequences *in vivo* (29–32). Investigations of DNA-binding dynamics on nonspecific DNA at the single-molecule level have significantly advanced our understanding of how proteins with diverse functions conduct their target DNA search (31,33,34). However, the paradoxical requirements of rapid search at nonspecific sites and stability at target sites have been primarily investigated in theoretical studies (35–38), direct comparisons of the protein-binding energy landscape at nonspecific sites and target sites from single-molecule experimental data are still lacking.

Here we used single-molecule fluorescence imaging to study the dynamics of quantum dot (QD)-labeled TRF1 and TRF2 proteins on λ DNA and DNA substrates containing alternating regions of telomeric and nontelomeric sequences. TRF1 appears to bind directly to telomeric sequences with very little 1D searching through nontelomeric DNA, whereas TRF2 possesses a significant component of 1D search. Using a truncation mutant, we localized this 1D searching activity to the basic domain of TRF2. On telomeric DNA both TRF1 and TRF2 diffuse slowly due to higher energy barriers to diffusion; and they possess longer attached lifetimes at telomeric repeats compared with nontelomeric DNA sequences. These observations indicate that there is preferential binding to telomeric DNA but the affinity is not high enough to prevent TRF proteins from diffusing along TTAGGG repeats. We postulate that this allows TRF1 and TRF2 to find their protein partners locally, and that this is a more general mechanism for coupling the energy from multiple weak DNA-binding components to ensure high binding specificity on long repetitive sequences.

## MATERIALS AND METHODS

### Protein purification

Recombinant N-terminal His_6_-tagged TRF1 and TRF2 were purified using a baculovirus/insect cell expression system and an AKTA Explorer FPLC (GE Healthcare) as described previously (39). TRF2ΔB was purified using a bacterial expression system (40). Protein concentrations were determined using the Bradford assay. Proteins used in this study are >90% pure based on SDS–PAGE and Coomassie staining. Proteins are active in binding to the telomeric DNA substrate containing three TTAGGG repeats based on electrophoresis mobility shift assays (EMSAs).

### DNA substrates

λ DNA was purchased from New England BioLabs. Other DNA substrates used in this study are shown in Figure 1B and Supplementary Figure S1. pSXneo(T2AG3) plasmid DNA containing 270 TTAGGG repeats was a gift from Dr Peter Lansdorp (University of British Columbia) (41). pGTK4 plasmid-derived Tel10 plasmid is 5994-bp long and contains 10 TTAGGG repeats and was prepared as described previously (42). To generate DNA fragments containing TTAGGG repeats for AFM imaging, digestion of T270 DNA (10 μg) was carried out at 37°C for 4 h using *HpaI* (130 U) in Buffer 4 (New England BioLabs). For Tel10 plasmid, digestions were carried out using *XbaI* (100 U) in Buffer 4. For fluorescence imaging, linearized plasmids were ligated to generate longer DNA substrates using a Quick Ligation™ Kit (New England BioLabs). The ligation reactions were done at room temperature for 15 min. The nontelomeric DNA substrate without the (TTAGGG)_270_ sequence was gel purified after the digestion of pSXneo(T2AG3) with *BglII* and *XbaI*. Final DNA substrate purification was done using an illustra GFX^TM^ PCR DNA and Gel Band Purification Kit (GE Healthcare).

### Protein–QD conjugation

Streptavidin-conjugated QDs (Sav-QDs) were purchased from Invitrogen. Biotinylated multivalent chelator tris-nitrilotriacetic acid (^BT^tris-NTA) was prepared according to the previous reports (43,44). The TRF–DNA reaction buffer contains 50 mM HEPES (pH 7.5) and varying concentrations of NaCl (25, 50, 75 and 100 mM). The total ionic strengths are 75, 125, 175 and 225 mM, respectively (45).

For single color QD labeling of His_6_-tagged TRF1 or TRF2, 1 μl of red QD (655 nm, 1 μM, Invitrogen, hydrodynamic radius: 11.5 nm) was incubated with 1 μl of ^BT^tris-NTA (2 μM) for 20 min (46). An amount of 1 μl of proteins (2 μM) were then added to the QD-NTA solution and incubated for additional 20 min. For dual-color QD labeling, 1 μl of red (1 μM) and green QDs (565 nm, 1 μM, hydrodynamic radius: 9.5 nm) were incubated with 1 μl of ^BT^tris-NTA (2 μM) (46). TRF1 or TRF2 (1 μl, 2 μM) was added to the solution and incubated for additional 20 min. For fluorescence imaging, unless otherwise specified, protein–NTA-QD solutions were diluted 200-fold before being drawn into the flow cell using a syringe pump (model SP260p, World Precision Instruments) at 300 μl/ml flow rate. The final protein concentration was 3.3 nM for both TRF1 and TRF2. Protein concentrations and ionic strengths of the buffer used in this study are comparable to physiological conditions (Supplementary Text). For AFM imaging of TRF2-QDs in the presence of monoclonal TRF2 antibody (Imagenex Corporation), the Ab:TRF2:NTA:QD ratio was 1:1:2:1 or 5:1:2:1, and reactions were carried out at room temperature for 30 min after the addition of antibodies.

### AFM imaging and image analysis

All DNA and protein samples were diluted 10-fold in 1× AFM buffer [25 mM NaOAc, 25 mM HEPES–KOH (pH 7.5) and 10 mM Mg(OAc)_2_] before deposition onto a freshly cleaved mica (SPI Supply). The samples were then washed with MilliQ water and dried under a stream of nitrogen gas. All images were collected in tapping mode using a MFP-3D-Bio AFM (Asylum Research). Pointprobe® PPP-FMR probes (Nanosensors) with spring constants at ∼2.8 N/m (nominal value) were used. All images were captured at a scan size of 1 μm × 1 μm, a scan rate of 1–2 Hz, and a resolution of 512 × 512 pixels. The position of TRF proteins on DNA was analyzed using the software from Asylum Research.

### Fluorescence imaging and analysis of fluorescence microscopy data

Fluorescence imaging was carried out with an inverted microscope (Nikon Ti-E) equipped with an encoded motorized stage, perfect focus system (PFS) and a Ti-TIRF E motorized illuminator unit. Fluorescence imaging was performed by excitation at 488 nm using a solid-state laser (20 mW Sapphire DPSS), a 100× objective with a numerical aperture of 1.49 (APO TIRF, Nikon) and 1.5× additional magnification. The laser power was controlled by using neutral density filters. The excitation beam was reflected into the objective through a TIRF filter set containing zt488rdc and ET500LP filters. For simultaneous imaging of green (565 nm) and red (655 nm) QDs, a dual view simultaneous imaging system (DV2, Photometrics) was used in combination with a T605LPXR dichroic beamsplitter (Chroma) and a band-pass filter ET655/40 m (Chroma). The images were captured using an electron multiplied (EM) CCD camera (iXon DU897, Andor Technology) operated at −60°C, with an EM gain of ∼250 and a frame rate of 20 Hz. Construction of the flow cell was carried out according to a procedure described previously (33,47,48). Silica beads (5 μm, Polysciences) were first treated with poly-l-lysine hydrobromide (2500 μg/ml, M.W. > 300 KDa, Wako Chemicals). λ DNA or ligated DNA substrate (5 μg/ml) were stretched, unless otherwise specified, under hydrodynamic flow at 300 µl/min flow rate using a syringe pump. Extended DNA strands anchored between two poly-l-lysine-coated beads formed DNA tightropes. After introducing the protein–QDs into the flow cell, all data collection was performed in the absence of any further buffer flow. The presence of YOYO-1 on DNA significantly reduced the diffusion constant, α-factor and the percentage of motile protein–QD complexes on DNA at certain salt conditions. Consequently, all data analysis was done using movies collected from using unstained DNA tightropes (Supplementary Text).

### Statistical analysis

Single-factor ANOVA and Student-*t* tests were used for statistical analysis.

## RESULTS

### TRF1- and TRF2-QD conjugates are functional in DNA binding

Fluorescent labeling of TRF1 and TRF2 was achieved by conjugating 6× histidine (His_6_) tagged TRF1 and TRF2 to streptavidin-conjugated QDs using the biotinylated multivalent chelator tris-nitrilotriacetic acid (^BT^tris-NTA) (44) (Figure 1B, see ‘Materials and Methods’ section). The multiple Ni-NTAs on the circular scaffold of the tris-NTA adaptor bind the His-tag with subnanomolar affinity, resulting in a bound lifetime in the range of hours (43,44). Importantly, we applied a previously established method based on AFM imaging to characterize the stoichiometry of QD–TRF complexes (49,50). AFM imaging revealed that using TRF2 antibody marking the presence of TRF2 (TRF2:Ab = 1:1 or 1:5), among the QDs displayed TRF2-Ab complexes (24%), 90% (*n* = 39) possessed only one TRF2–Ab complex (Supplementary Figure S2).

QDs alone exhibited minimal nonspecific binding to DNA as confirmed by AFM (Figure 1C). As expected, addition of QD-labeled TRF1 or TRF2 to DNA containing two stretches of (TTAGGG)_135_ connected by a short linker region (T270 DNA, Figure 1B, see ‘Materials and Methods’ section) resulted in substantial binding (Figure 1D and E). Furthermore, AFM image analysis revealed that both TRF1- and TRF2-QDs bound preferentially to the telomeric DNA sequences on both the T270 and Tel10 DNA substrates (Supplementary Figure S3).

### TRF1 and TRF2 diffuse one-dimensionally on nontelomeric DNA

**Figure 2.**
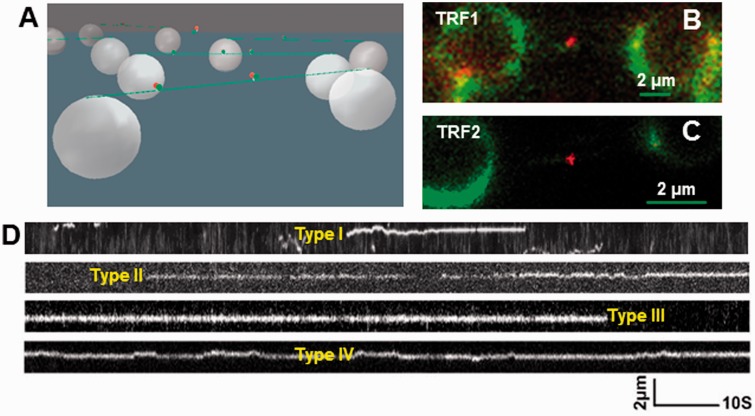
DNA tightrope assay based oblique-angle fluorescence imaging of TRF1- and TRF2-QDs on λ DNA tightropes. (**A**) A schematic drawing of the DNA tightropes (green lines) bound with QD (red ball)-labeled proteins (green balls) between silica beads (large white balls). The drawing is not to scale. (**B** and **C**) Representative fluorescence images of red (655 nm) QD-conjugated His_6_-TRF1 (**B**) and His_6_-TRF2 (**C**) on λ DNA (stained with YoYo1). (**D**) Classification of different types of protein–DNA interactions observed with TRF2-QDs on λ DNA for attached lifetime measurement.

To study the dynamics of individual TRF1 and TRF2 molecules on DNA using oblique-angle fluorescence microscopy, we applied a DNA tightrope assay (Figure 2A) (33). DNA strands are suspended between poly-l-lysine coated microspheres at an elongation of ∼90% DNA contour length using hydrodynamic flow (47). This process isolates DNA from the surface and does not require continuous buffer flow for the observation of protein–DNA interactions. QDs did not bind to DNA tightropes alone or in the presence of TRF proteins without ^BT^tris-NTA. However, with both ^BT^tris-NTA and His_6_-tagged TRF1 or TRF2, QDs were observed on DNA throughout the visual field (Figure 2B and C). Both TRF1- (Supplementary Movie S1) and TRF2-QDs (Supplementary Movie S2) showed clear 1D diffusion on DNA, which was tracked by Gaussian fitting to kymographs (particle position versus time plots, Supplementary Data) (33,47).

To determine whether TRF1 and TRF2 slide or hop, we evaluated the effect of ionic conditions on the dynamic interactions between the QD-labeled TRF proteins and DNA. Increasing the salt concentration should not affect the diffusion constants of a sliding process, but should elevate the diffusion constants of hopping (29,51,52). We performed experiments at 75, 125, 175 and 225 mM ionic strengths (see ‘Materials and Methods’ section). The fraction of motile TRF1 proteins ranged from 15% to 33% (Supplementary Figure S4A) and followed a trend of decreasing diffusion constants as the ionic strength increased (7.5–3.8 × 10^−^^2 ^µm^2^/s), such that the difference between the highest and lowest salt was statistically significant (*P* = 0.017; Supplementary Figure S4B and Table 1). In contrast, TRF2 was highly motile on λ DNA across all ionic strengths and showed no significant change in diffusion constant (8.4–9.5 × 10^−^^2^ µm^2^/s, Supplementary Figure S4B and Table 1). TRF2 diffused substantially faster than TRF1 at all ionic strengths showing statistical significance at ionic strengths between 125 and 225 mM.

**Table 1.** Summary of the diffusion constant, α factor, and lifetime of Sav-QD (655 nm)-labeled TRF1 and TRF2, on λ DNA, at different ionic strengths

Ionic Strength (mM)	TRF1			TRF2		
	D (×10^−2^µm^2^/s)	α Factor	Lifetime (s)	D (×10^−2^µm^2^/s)	α Factor	Lifetime (s)
75	7.5 ± 1.2 (51)	0.65 ± 0.04 (51)	–	8.9 ± 0.9 (59)	0.94 ± 0.05 (59)	10 ± 0.1 (104)
125	5.5 ± 1.4 (37)	0.72 ± 0.05 (37)	–	8.4 ± 0.9 (54)	0.95 ± 0.06 (54)	2.5 ± 0.1 (106)
175	4.9 ± 1.0 (40)	0.72 ± 0.06 (40)	1.8 ± 0.1(63)	9.5 ± 0.1 (63)	0.82 ± 0.03 (63)	4.6 ± 0.1 (107)
225	3.8 ± 1.2 (33)	0.89 ± 0.07(33)	0.3 ± 0.01(128)	9.5 ± 0.1 (66)	0.84 ± 0.04 (66)	3.4 ± 0.1 (95)
125- TRF2ΔB				9.1 ± 1.8 (21)	0.93 ± 0.04 (21)	–

The numbers in the parentheses indicate the total number of complexes analyzed. Lifetime was measured for complexes showing both protein binding and release events within the video frame (Type I, Figure 2D). Data are presented as mean ± standard error.

In addition to the diffusion constant, we also measured the diffusive exponent (α-factor, Supplementary Data). An α factor of 1 indicates an unbiased random walk, >1 indicates directed motion and <1 indicates periods of pausing in the random walk (subdiffusion) (53). TRF1 showed a slight trend toward increasing α factor from 0.65 to 0.89 with increasing ionic strength (Supplementary Figure S4C and Table 1); this result suggests pausing at low ionic strength, which is abrogated by salt. For TRF2, however, the α factor was consistently ∼1 and did not show any significant variation with ionic strength, suggesting an unbiased random walk. Dual-color labeling of the TRF proteins allowed us to assess whether protein hopping could enable bypass of other DNA-bound proteins that act as diffusion barriers (Supplementary Figure S5). Neither TRF1 nor TRF2 could bypass differentially labeled proteins of the same species on DNA, which is consistent with a TRF2 sliding mechanism and suggests that TRF1 also navigates DNA by sliding (Supplementary Data).

Next, we measured the attached lifetimes of protein–QD complexes on DNA. First however, we classified the protein–DNA interactions into four types based on how they behaved during a movie. Type I: protein binds and then releases; Type II: proteins binds and doesn’t leave; Type III: protein is bound at the beginning of the movie but releases; Type IV: protein is bound from the beginning to end of the movie (Figure 2D and Supplementary Table S1). Reliable attached lifetime measurements could only be obtained from analysis of the Type I interactions. The lifetimes of both TRF1 and TRF2 on λ DNA decreased with increasing ionic strength, ranging from 1.8 s (175 mM) to 0.3 s (225 mM) for TRF1 and from 10 s (75 mM) to 3.4 s (225 mM) for TRF2 (Table 1 and Supplementary Figure S4D). These results are consistent with salt-sensitive electrostatic interactions between TRF proteins and DNA and increased probability of dissociation from DNA during sliding as the ionic strength increases (54).

In summary, these results demonstrate that both TRF1 and TRF2 slide on DNA in search of their target DNA-binding sites. TRF2 is a canonical slider, whereas TRF1 also appears to slide but may alter its conformation with salt.

### TRF1 and TRF2 bind specifically to telomeric sequences on DNA tightropes

**Figure 3.**
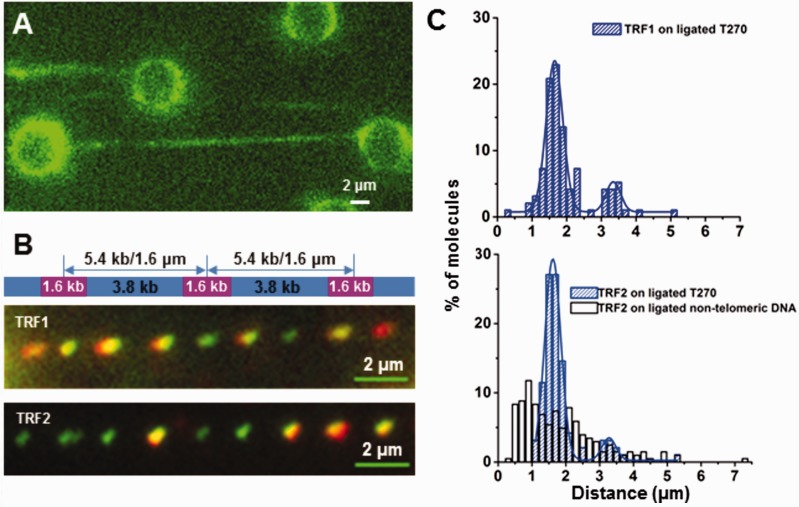
TRF1- and TRF2-QDs bind specifically to telomeric sequences on DNA tightropes. (**A)** A representative fluorescence image of DNA tightropes formed using ligated linear T270 DNA containing telomeric sequences (stained with YoYo1). (**B**) A schematic drawing of the ligated T270 DNA substrate (top) and representative fluorescence images of dual color (655 and 565 nm)-labeled TRF1- (middle) and TRF2-QDs (bottom) on the ligated T270 DNA substrate. (**C**) Measured distances between two adjacent TRF1- (*n* = 96, top) and TRF2-QDs (bottom, *n* = 96) on the ligated T270 substrate (blue bars), and between TRF2-QDs on the nontelomeric DNA substrate (bottom, white bars, *n* = 204). The lines in the top and bottom panels are double Gaussian fits to the data, which have *R*^2^ of 0.99 and 0.95, respectively.

To examine the dynamics of TRF1 and TRF2 binding to telomeric DNA sequences, we ligated linearized T270 DNA to generate long DNA substrates with alternating (TTAGGG)_270_ telomeric and nontelomeric regions (Figure 3A). The lengths of these DNA tightropes ranged from ∼2.1 to ∼22 μm, consistent with ligation of 2–12 of 5.4 kb T270 DNA fragments (Supplementary Figure S6A). TRF1 and TRF2 bound to the ligated T270 DNA tightropes with regular spacing (Figure 3B, Supplementary Movies S3 and S4). For both TRF1 and TRF2, the distributions of the distances between adjacent binders fit well to the sum of two Gaussian distribution functions centered at ∼1.6 and 3.2 μm (Figure 3C). These findings are consistent with the expected spacing of the telomeric regions (Figure 3B). In contrast, on the ligated nontelomeric DNA, the distribution of TRF2 spacing was broad (Figure 3C), and no examples of three or more bound protein–QDs on individual DNA tightropes with a spacing of ∼1.6 or 3.2 μm were observed for either TRF1 or TRF2. As an additional control the telomeric repeats were spaced further apart using a 5.99-kb long DNA substrate containing only 10 TTAGGG repeats (Tel10, Supplementary Figure S7 and Supplementary Movie S5) and, as expected, adjacent bound TRF2 molecules were further apart (1.9 μm, ∼95% contour length) than on T270.

**Figure 4.**
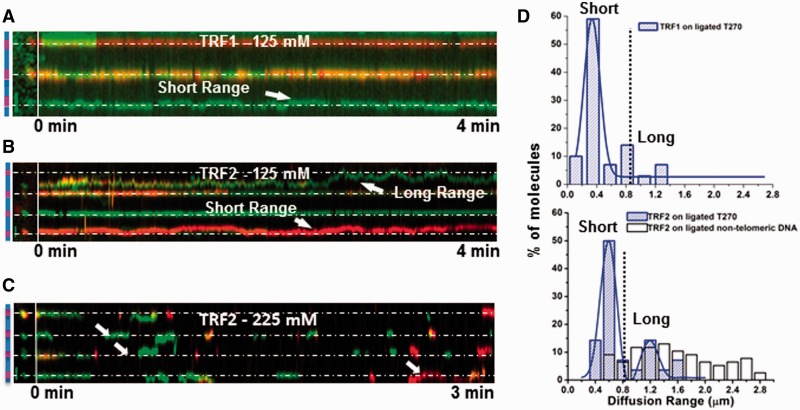
TRF1 and TRF2 show different diffusional properties over telomeric region versus nontelomeric regions. (**A–C**) Kymographical analysis of dual color (655 and 565 nm)-labeled TRF1 (**A***,* 125 mM ionic strength) and TRF2 (**B**:125 and **C**:225 mM ionic strengths) on the ligated T270 DNA. The panel left to the vertical white line shows a schematic drawing of the ligated T270 substrate with telomeric (purple) and nontelomeric sequences (blue), and a fluorescence image of the DNA with protein–QDs. The horizontal white lines indicate the estimated center of the telomeric region based on the spacing between adjacent QDs. The white arrows in (C) indicate TRF2 diffusing between two adjacent telomeric sequences. (**D**) The diffusion range distributions of TRF1-(top, *n* = 29) and TRF2-QDs (bottom, *n* = 28) on the ligated T270 substrate (blue bars), and TRF2-QDs on the nontelomeric DNA (bottom, white bars, *n* = 77). Diffusion ranges below and beyond 850 nm are categorized into short (telomeric) and long range (nontelomeric), respectively. The lines in the top and bottom panels of (D) are single and double Gaussian fits to the data, respectively, which have *R*^2^ of 0.90 and 0.96, respectively.

We also examined how far single molecules of TRF1 and TRF2 could slide on the ligated nontelomeric DNA versus ligated T270 DNA (Figure 4, Supplementary Movies S3 and S4). On T270 DNA, TRF1 displayed one major population with diffusion ranges centered on 0.38 µm (Figure 4D). TRF2 exhibited two distinct populations centered on 0.5 and 1.2 μm at 125 mM ionic strength, and on 0.5 and 1.5 μm at 225 mM ionic strength (Figure 4 and Supplementary Figure S6B). But on nontelomeric DNA, no clear peak was evident (white bars, Figure 4D). Approximately 90% (*n* = 29) of TRF1 and 73% (*n* = 30) of TRF2 diffused in a short range (<850 nm). The diffusion range was invariant across all time windows (∼10–100 s, Supplementary Figure S8), ruling out the possibility that the short range diffusion observed was due to shorter video lengths. Instead, this finding suggests that once the molecules are within a telomeric region, they tend to remain there. We explored the possibility that short range diffusion was caused by multiple proteins binding to the same telomeric region and restricting 1D sliding. However, at a lower TRF2 concentration, the short diffusion range did not change (compare Figure 4D and Supplementary Figure S6C). Therefore, the two diffusion range populations could be assigned to diffusion of TRF proteins over the (TTAGGG)_270_ telomeric regions (0.5 μm, ∼90% contour length) and the nontelomeric spacers (1.2 μm, ∼90% contour length), respectively (Figure 3B). For TRF2, transitions were observed between telomeric and nontelomeric regions or even between two adjacent T270 repeats, which were more frequent at 225 mM ionic strength (white arrows, Figure 4C). These events provided the peak with diffusion range centered at ∼1.5 µm (Supplementary Figure S6B).

Taken together, the regular spacing between QD-labeled TRFs demonstrated that TRF1 and TRF2 bind specifically to the telomeric regions on both T270 and Tel10 DNA substrates. These results also showed that compared with TRF2, TRF1 undergoes a greater number of direct binding events from solution to the (TTAGGG)_270_ region, forgoing a 1D search (Figure 4D).

### TRF1 and TRF2 exhibit slower dynamics on telomeric DNA

To quantify the diffusion constants at the (TTAGGG)_270_ telomeric region, we selectively analyzed TRF1 and TRF2 on the ligated T270 DNA tightropes with at least three or more protein–QDs in a row spaced at the length of nontelomeric spacers (1.5–1.7 μm, Figures 3 and 4). TRF1 and TRF2 diffused at ∼0.15–0.22 × 10^2^ µm^2^/s and 0.27–0.29 × 10^−^^2^ µm^2^/s at the (TTAGGG)_270_ region, respectively. These rates are ∼17- to 37- and ∼30-fold slower, for TRF1 and TRF2, respectively, compared with those on λ DNA at the same ionic strength (Tables 1 and 2). We noted that in many cases TRF proteins binding to telomere repeats would be confined to diffuse within this region due to the higher affinity for telomeric sequences (Figure 4 and Supplementary Figure S8). To ensure that this confinement would not artificially reduce the apparent diffusion constant, we simulated 1D diffusion of proteins on a linear DNA lattice of unlimited length versus a 1.6 kb total length, which mimics the (TTAGGG)_270_ region (Supplementary Text). These simulations revealed that confinement within 1.6 kb DNA does not significantly reduce the observed diffusion constant at the (TTAGGG)_270_ region (Supplementary Figure S9). In addition, camera-based time-averaging was not a major contributor to the observed slower diffusion constants at the telomeric region under these experimental conditions (Supplementary Data). An alternative fitting method to simultaneously determine the diffusion constant and confined DNA length also provided similar results (Supplementary Data) (55). Furthermore, the diffusion constants of TRF2-QDs (0.31 ± 0.003 × 10^−^^2 ^µm^2^/s, n = 37) on DNA tightropes formed under a 12× slower flow rate (25 μl/min) are not significantly different from those on DNA tightropes stretched at a higher flow rate (300 μl/min) (Table 2). Under this condition, DNA tightropes were under less tension with final extension to only ∼88% of DNA contour length (Supplementary Figure S6D). These results suggest that under these conditions, diffusion constants of TRF2 do not vary significantly with the amount of tension on dsDNA tightropes.

**Table 2.** Summary of the diffusion constant and lifetime of TRF1- and TRF2-QDs on the ligated T270 DNA substrates

DNA	Ionic strength (mM)	TRF1				TRF2			
		Telomeric		Nontelomeric		Telomeric		Nontelomeric	
		D ×10^−2 ^µm^2^/s	Lifetime (s)	D ×10^−2 ^µm^2^/s	Lifetime (s)	D ×10^−2 ^µm^2^/s	Lifetime (s)	D ×10^−2 ^µm^2^/s	Lifetime (s)
Tel270	125	0.15 ± 0.02 (22)	17.3 ± 0.2 (15)	1.0 ± 0.2 (8)	5.9 ± 0.2 (6)	0.27 ± 0.04 (22)	14.8 ± 0.2 (105)	3.0 ± 0.5 (8)	4.4 ± 0.1 (53)
Tel270	225	0.22 ± 0.04 (21)	9.2 ± 0.2 (9)	1.8 ± 0.7 (5)	5.7 ± 0.3 (2)	0.29 ± 0.04 (34)	10.3 ± 0.6 (115)	9.9 ± 3.0 (7)	3.8 ± 0.3 (41)
Tel10	125					–	6.7 ± 0.4 (50)	–	3.3 ± 0.2 (51)

Proteins were labeled with equal molar amount of red (655 nm) and green (565 nm) QDs. The numbers in the parentheses indicate the total number of complexes analyzed. Lifetime was measured for complexes showing both protein binding and release events within the video frame (Type I, Figure 2D). Data are presented as mean ± standard error.

We observed that TRF1 and TRF2 can directly dissociate from telomeric regions or through nontelomeric regions (Supplementary Figure S10). Overall, we found that the relative proportions of Type I (protein binds and releases) and Type IV (protein is bound from the beginning to end of the movie) protein–DNA interactions observed during the experimental time course depended on the DNA substrate (Supplementary Table S1). For TRF1 on T270 DNA the vast majority of molecules were Type IV, indicating a considerably longer attachment. Consistent with this result, the average lifetime of Type I TRF1 bound to the telomeric sequences on T270 DNA was ∼31-fold longer than that for λ DNA (9.2 s versus 0.3 s, 225 mM ionic strength, Tables 1 and 2). TRF2 behaved quite differently, showing a less pronounced difference between the proportions of Type I and Type IV complexes on λ DNA and T270 DNA. Furthermore, the attached lifetimes for Type I TRF2 complexes was only ∼3-fold longer at the telomeric regions on T270 DNA compared with λ DNA (10.3 s versus 3.4 s: Tables 1 and 2). It is worth noting that the lifetimes of TRF proteins on DNA are longer than the QD blinking rate (56), ruling out artifacts from QD blinking in the lifetime measurement. In summary, compared with binding to nontelomeric DNA, both TRF1 and TRF2 possess distinctly slower detachment and diffusional dynamics on the telomeric DNA.

### The basic domain is essential for the 1D search by TRF2

The basic domain at the N-terminus of TRF2 permits its binding to model replication forks and four-way junctions independent of telomere sequences (57). In addition, the absence of this domain leads to a diminished ability of TRF2 to localize to model telomere ends and to facilitate T-loop formation (57). We created and imaged a basic domain deletion mutant of TRF2 (TRF2ΔB) on λ DNA and the ligated T270 (Supplementary Figure S11). Compared with full-length TRF2, TRF2ΔB-QDs have higher specificity for the telomeric sequences on T270 DNA substrate and lower affinity to DNA ends (compare Supplementary Figures S11A and S3B). Furthermore, relative to the full-length TRF2, the fraction of motile protein–DNA complexes decreased by ∼1.5-fold for TRF2ΔB (Supplementary Figure S11 legend). Interestingly, the diffusion constant (9.1 ± 1.8 × 10^−^^2^ µm^2^/s) and α-factor (0.93 ± 0.04) of TRF2ΔB on λ DNA were not significantly different from those of full-length TRF2 (Table 1). However, the percentage of complexes undergoing long-range diffusion (10% at 125 mM ionic strength) was significantly lower (*P* = 0.01) than for full-length protein (27%) at the same ionic strength (Supplementary Figure S11D). On T270 DNA, majority of motile TRF2ΔB (90%) was found with a diffusing range consistent with length of the telomeric region on T270 DNA, suggesting that TRF2ΔB directly associates with telomeric DNA from solution and not by diffusion from a nontelomeric region. Since the frequency of TRF2ΔB DNA binding was lower than the full-length protein (1.1 versus 3.8 molecules/bead pair), it was not possible to restrict the analysis to those tightropes with three adjacent bound molecules. Therefore, we treated all short range diffusion (<850 nm) by TRF2ΔB on the ligated T270 as diffusion over the telomeric region. The dynamics of TRF2ΔB over the (TTAGGG)_270_ region were similar to those of full-length TRF2, with a similar diffusion range (0.47 ± 0.03 µm, Supplementary Figure S11D) and diffusion constant (0.27 ± 0.01 × 10^−^^2^ µm^2^/s at 125 mM and 0.26 ± 0.01 × 10^−^^2 ^µm^2^/s at 225 mM). These observations suggest that the basic domain of TRF2 normally facilitates its 1D search on nontelomeric DNA. The reduced degree of TRF2 localization to the telomeric region due to deletion of the basic domain demonstrates the importance of 1D diffusion in the TRF2 telomeric target site search (Supplementary Figure S11).

## DISCUSSION

TRF1 and TRF2 are the only scaffolding shelterin proteins that bind directly to duplex telomeric DNA. The results presented here from single-molecule imaging of TRF1 and TRF2 dynamics on telomeric and nontelomeric DNA provide for the first time a fundamental understanding of the mechanisms that drive the dynamics of shelterin assembly/disassembly at telomeres.

### TRF2 performs 1D searching more effectively than TRF1 to find telomeric sequences

**Figure 5.**
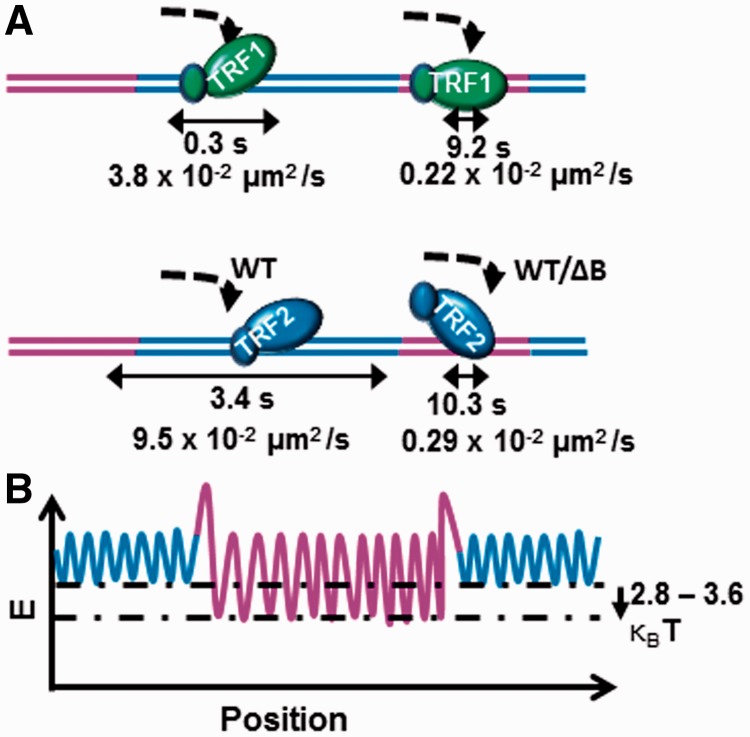
TRF1 and TRF2 strike a balance between target search and specificity. (**A**) TRF1 and TRF2 can undertake a 1D search on DNA consistent with rotation-coupled diffusion along the DNA helix. The small ovals represent the basic and acidic domains of TRF1 and TRF2. The blue and purple lines represent nontelomeric and telomeric DNA, respectively. TRF1 relies more on 3D search and majority of the TRF2ΔB molecules bind to the telomeric region directly from solution forgoing the 1D component of the search. (**B**) The energy landscape along the positions at telomeric and nontelomeric sequences. The diffusion constant and lifetime measurements are consistent with ∼2.8–3.6 κ_B_T higher energy barriers to diffusion at the telomeric sequences in comparison with nontelomeric sequences (Tables 1 and 2). The additional energy barrier at the nontelomeric and telomeric junction represents the activation energy needed for conformational change/DNA-binding domain switching on proteins to achieve specific binding.

Rotational tracking along DNA during which a protein follows a helical track along the DNA to maintain optimal contact has been inferred for several DNA-binding proteins (58). The measured diffusion constants for TRF1 and TRF2 obtained using the DNA tightrope assay were consistent with rotational tracking of the DNA helix (Table 1 and Supplementary Text), although slightly higher than the predicted upper limit for this motion (2.1 × 10^−^^2^ µm^2^/s, Supplementary Text). This discrepancy could be due to the flexible linkage mediated by the His-tag and ^BT^tris-NTA between TRF proteins and QDs (59). The measured diffusion constants together with the lack of observed barrier bypass events in dual color experiments (Supplementary Figure S5) demonstrated that both TRF1 and TRF2 track the DNA helix to maintain optimum contact between their DNA-binding surfaces and the DNA (Figure 5A). However, the attached lifetime of Type I TRF1 at the nontelomeric region was 10-fold shorter than that of TRF2 (0.3 s versus 3.4 s at 225 mM, Table 1). These results are consistent with a significantly lower percent of TRF1 molecules exhibiting long range diffusion compared with TRF2 (Figure 4). This difference between TRF1 and TRF2 is partly due to the sequences at the N-termini of TRF proteins (Figure 1A). For TRF2 this region contains a basic domain, the deletion of which (TRF2ΔB) led to a clear reduction in the percentage of motile protein complexes on λ DNA. Importantly, it was observed that 90% of TRF2ΔB molecules underwent short-range diffusion consistent with the length of the telomeric regions (Supplementary Figure S11). This result suggests that the majority of the TRF2ΔB molecules found the telomeric region directly from solution, forgoing the 1D component of the search (Figure 5A). These results support the notion that domain B facilitates the association of TRF2 to nonspecific DNA and this results in sliding subsequently. However, the diffusion constant and α factor of TRF2ΔB were not significantly different from the full-length protein (Table 1). We speculate that TRF2ΔB containing the Myb-type domain has weak DNA-binding affinity for nontelomeric DNA. On nontelomeric λ DNA, the DNA-binding energy landscapes are similar for full-length TRF2 and TRF2ΔB, leading to similar diffusion constants. However, it is unclear whether in full-length TRF2, nonspecific DNA binding is solely dependent of domain B or combination of this domain and the Myb domain. TRF1 behaved similarly to TRF2ΔB, perhaps as a consequence of also lacking the basic domain. Therefore, unlike TRF1, TRF2 can bind to nontelomeric sequences and use a 1D search to more efficiently locate telomeric DNA.

### Comparing the 1D diffusion of TRF1 and TRF2 on nontelomeric and telomeric DNA

We found that, in general, TRF2 slides faster than TRF1 at nontelomeric sequences (Supplementary Figure S4B and Table 1). The diffusive exponent was <1 only for TRF1 at lower ionic strengths, consistent with subdiffusive motion or pausing during diffusion (Supplementary Figure S4C and Table 1). Together, these observations indicate that TRF2’s diffusion is consistent with the canonical description of sliding. However, TRF1’s behavior changed with salt in a manner that was inconsistent with a solely electrostatic-mediated protein–DNA interaction (60), and suggesting a possible conformational rearrangement induced by salt at the DNA-binding interface. This rearrangement could lead to obstacles to diffusion and/or traps within the binding energy landscape or escape time (53).

The Myb type DNA-binding domain of TRF2 has a 4-fold weaker DNA-binding affinity than the Myb domain in TRF1 (equilibrium dissociation constants *K*_d_: 750 versus 200 nM, respectively) (61). The diffusion constant of TRF1 was ∼2-fold slower than that of TRF2 within telomeric repeats (125 mM ionic strength, Table 2). This result is equivalent to ∼0.6 κ_B_T increase in the roughness of the DNA-binding landscape or ∼2-fold change in affinity. While these results are consistent with the stronger binding to the telomeric sequences by TRF1 Myb domain, other domains on TRF proteins could also indirectly influence the DNA-binding dynamics of these two proteins over the telomeric regions. Furthermore, the difference in the dynamics of the TRF proteins between telomeric DNA and nontelomeric DNA is due to inherent sequence effects and therefore likely represents the situation *in vivo*. This is further supported by the ionic conditions used in our experiments which were chosen to represent those encountered *in vivo* (Supplementary Text).

### TRF1 and TRF2 strike a balance between search and specificity

TRF proteins face a unique challenge. They must find both their cognate sites and protein partners to form the shelterin complex, and to regulate the functions of a myriad of proteins involved in telomere maintenance and cell-cycle progression (26). For example, TRF1 and TRF2 both bind to TIN2 to form a ternary complex of TRF1, TRF2 and TIN2 (27,28). Importantly, TRF2 is a protein hub interacting with several DNA-binding proteins that play important roles in DNA repair, including WRN, Ku70-Ku80 and ERCC1-XPF (26,39,62,63). This requires that TRF proteins retain specificity for their DNA target site but also the ability to slide within the telomeric regions to encounter protein partners to form protein complexes.

The binding energy of a protein along DNA contains a series of local energy minima separated by energy barriers. Protein sliding on DNA has been modeled as a particle diffusing along a rough potential energy landscape. The roughness of the landscape reduces the diffusion constant from the theoretical maximum determined by solution viscosity. We found that the diffusion of TRF1 and TRF2 was ∼17- to 37-fold slower at telomeric regions compared with nontelomeric λ DNA, corresponding to ∼2.8–3.6 κ_B_T increase in the roughness of the energy landscape (Supplementary Text and Figure 5B). Also, the TRF1 and TRF2 attached lifetimes within telomeric sequences were ∼31- and 3-fold longer, respectively, compared with those on λ DNA (225 mM ionic strength, Table 2). These differences correspond to an increase of ∼3.4 κ_B_T (for TRF1) and 1.1 κ_B_T (for TRF2) in relative binding energy at the telomeric regions (Supplementary Data). Taken together, the relative activation energy barriers based on the diffusion constants and lifetimes are not only consistent with each other, but also close to the estimated minimal roughness of the energy landscape at specific binding sites (∼6.6 κ_B_T) for a genome size of 3 × 10^9 ^bp (Supplementary Data) (35).

Interestingly, the percentage of TRF2 arriving at the (TTAGGG)_270_ region (73%, 125 mM; Figure 4D) was lower than the simulated equivalent situation assuming TRF2 first binds to the nontelomeric spacer (98%, *n* = 500). This discrepancy is consistent with an additional activation energy barrier between telomeric and nontelomeric regions, likely due to a switch within TRF2 from a nonspecific binding mode to a specific recognition mode (Figure 5B) (64). Noticeably, for TRF2, this energy barrier was lower at 225 mM ionic strength than at 125 mM, since more proteins arrived at telomeric regions from the nontelomeric spacers (Figure 4D and Supplementary Figure S6B), consistent with the desolvation of electrostatic residues required for DNA binding.

In contrast to the metastable and dynamic nature of the TRF protein binding to telomeric sequences (Figure 5), other systems characterized by single-molecule imaging show long-lived stable binding to specific sequences. For example, the mismatch repair protein, MutSα binds to a mismatch (+ADP) with a half-life of 9.6 ± 1.5 min (36); and the average lifetime of the Type III restriction enzyme EcoP15I on DNA with specific binding sites was ∼180 s (38). The primary differences between these systems are the target DNA sites. For TRF proteins, the target is a long repetitive sequence, whereas for other systems target sites consist of much shorter nonrepetitive DNA. We propose that TRF proteins utilize the combined free energy of binding from the association of multiple TRF proteins in the same region to increase binding specificity and stability. For example, TRF1 and TRF2 linked by TIN2 would increase the total affinity for telomeric sequences by summing the interaction energies of TRF1 and TRF2. We postulate that *in vivo* the diffusional properties of TRF proteins at the telomeric regions enable these proteins to search for their protein partners, such as another TRF–TIN2 complex, to assemble stable shelterin complexes on telomeric substrates. In this putative model of partner search, we expect that long distance searching is unlikely due to DNA-bound obstacles such as nucleosomes and other DNA-binding proteins. Rather 1D diffusion represents a relatively local search mechanism which increases the probability of partner encounter during the attached period. In cells the intrinsic dynamics of TRF1 and TRF2 could potentially be important for regulating the assembly and disassembly of shelterin complexes, and switching between different telomere structures (capped and uncapped states).

In summary, using QD-conjugated proteins, DNA tightropes embedded with site-specific sequences, AFM and fluorescence imaging, we reveal that TRF1 and TRF2 use different mechanisms to find telomeric DNA but share a novel mechanism to search for protein partners at telomeres. Based on these results, we postulate a general mechanism for how multiprotein complexes strike a balance between achieving specificity and target search, in a process we define as ‘tag-team proofreading’. In this model, proteins first form weak transient complexes with their cognate DNA sequences, and then rely on the additive energies of binding provided by partner proteins to generate higher specificity.

## SUPPLEMENTARY DATA

Supplementary Data are available at NAR Online, including [65–73].

## FUNDING

The BBSRC [BB/I003460/1 to N.M.K.]; National Institutes of Health [ES0515052 to P.L.O. and 4R00ES016758 to H.W.]. Funding for open access charges: National Institutes of Health [4R00ES016758].

*Conflict of interest statement*. None declared.

## Supplementary Material

Supplementary Data
