# Supplementary material for: TRF1 and TRF2 use different mechanisms to find telomeric DNA but share a novel mechanism to search for protein partners at telomeres
Source: Nucleic Acids Res. 2013 Nov 22;42(4):2493–504. doi: 10.1093/nar/gkt1132 (PMC3936710; doi:10.1093/nar/gkt1132)
Supplement: Supplementary Data [file supp_42_4_2493__index.html]

TRF1 and TRF2 use different mechanisms to find telomeric DNA but share a novel mechanism to search for protein partners at telomeres — TRF1 and TRF2 use different mechanisms to find telomeric DNA but share a novel mechanism to search for protein partners at telomeres — Supplementary Data 

# TRF1 and TRF2 use different mechanisms to find telomeric DNA but share a novel mechanism to search for protein partners at telomeres

## Supplementary Data

files

**Files in this Data Supplement:**

- Supplementary Data - pdf file
- Supplementary Data - avi file
- Supplementary Data - avi file
- Supplementary Data - avi file
- Supplementary Data - avi file
- Supplementary Data - avi file
