# Supplementary material for: TRF1 and TRF2 use different mechanisms to find telomeric DNA but share a novel mechanism to search for protein partners at telomeres
Source: Nucleic Acids Res. 2013 Nov 22;42(4):2493–504. doi: 10.1093/nar/gkt1132 (PMC3936710; doi:10.1093/nar/gkt1132)
Supplement: Supplementary Data [file supp_gkt1132_nar-02791-m-2013-File007.pdf]

SUPPLEMENTARY INFORMATION

For manuscript

TRF1 and TRF2 use different mechanisms to find telomeric DNA but share a novel mechanism to search for protein partners at telomeres

Jiangguo Lin[1], Preston Countryman[1], Noah Buncher[2], Parminder Kaur[1], Longjiang E[3], Yiyun Zhang[4], Greg Gibson[5], Changjiang You[6], Simon C. Watkins[5], Jacob Piehler[6], Patricia L. Opresko[2], Neil M. Kad[7,*], Hong Wang[1,*]

[1]Physics Department, [4]Industry and System Engineering, North Carolina State University, Raleigh, North Carolina, NC 27695, USA

[2]Department of Environmental and Occupational Health, [5]Center for Biologic Imaging, University of Pittsburgh Graduate School of Public Health, Pittsburgh, Pennsylvania 15219, USA

[3]Electric and Computer Engineering, University of North Carolina at Charlotte, Charlotte, North Carolina 28223, USA

[6] Division of Biophysics, *Universität Osnabrück*, Barbarstrasse 11, 49076, *Osnabrück*, Germany

This supplementary information contains:

Supplementary Text
Supplementary Legends for Movies S1–5
Supplementary References
Supplementary Table S1
Supplementary Figures S1–11

SUPPLEMENTARY TEXT

Comparison between *in vivo* and *in vitro* conditions

Previously, HeLa cell lines (volume ~ 2000 μm^3) examined by Takai *et al.* contained approximately 0.4 to 1 x 10^5 molecules of TRF1 and TRF2 proteins per cell, corresponding to ~32 to 83 nM concentrations inside cells (65). However, given non-telomeric roles of TRF1 and TRF2, the numbers of TRF1 and TRF2 binding to the telomeric region are uncertain. The final concentrations of TRF proteins in our flow cells is 3.3 nM, which is at the upper limit of the concentration that we can use without generating too much background signal.

The physiological ionic strength is between 100 to 200 mM KCl or NaCl. In this study, we used total ionic strengths at 75 mM (with 25 mM NaCl) to 225 mM (with 100 mM NaCl) for investigating the dynamics of TRF proteins on telomeric and non-telomeric DNA.

Single-particle tracking

The resolution of tracking of proteins on DNA was determined to be 16 nm using the data from static TRF2-QDs binding to the $(TTAGGG)_{10}$ regions on ligated Tel10 DNA tightropes (Supplementary Figure S7) based on the following equation (66):

$$\sigma = \sqrt{\frac{s^2 + a^2/12}{N} + \frac{4s^3 b^2 \sqrt{\pi}}{aN^2}}$$

Equation 1

where N, a, b and s are the numbers of photons, the effective pixel size (110 nm), standard deviation of the background signal, and the standard deviation of 2D Gaussian fit, respectively.

The mean square displacement (MSD) as a function of time interval is given by:

$$MSD(n\Delta t) = \frac{1}{N-n}\sum_{i=1}^{N-n}\left[(x_{i+n} - x_i)^2 + (y_{i+n} - y_i)^2\right]$$

Equation 2

where N is the total number of frames in the trajectory, n is the number of frames for different time intervals, Δt is the time between frames, and x_i and y_i are the positions of the protein-QD in the frame *i.* The 1-D diffusion constant (*D*) and diffusion exponent (α factor) were analyzed by a custom routine developed in LabView Software based on the following equation (53):

$$MSD = 2Dt^{\alpha}$$

Equation 3

TRF2 binding at $(TTAGGG)_{10}$ sequence on the ligated Tel10 DNA substrate displayed an apparent diffusion constant of 2.5 (±0.05) ×10^{-4} μm^2/s (n = 10). This provides a baseline for the characterization of static complexes on DNA. A protein was categorized as being motile if the diffusion constant is greater than 5 ×10^{-4} μm^2/s and R^2 value from data fitting using Equation 3 is greater than 0.8.

For confined diffusion at the telomere sequences, an alternative equation was used to fit the MSD vs. Δt to determine the diffusion constant and confined length (55):

$$\langle x^2 \rangle(t) = \frac{L_x{}^2}{6} - \frac{16 L_x{}^2}{\pi^4} \sum_{n=1(odd)}^{\infty} \frac{1}{n^4} exp\left\{-\frac{1}{2}\left(\frac{n\pi\sigma_x}{L_x}\right)^2 t\right\}$$ **Equation 4**

where L_x is the confined length, and σ = 2D.
Data fitting using this model provides similar diffusion constants of 0.22 (±0.05) $\times 10^{-2}$ $\mu m^2/s$ and 0.37 (±0.05) $\times 10^{-2}$ $\mu m^2/s$ for TRF1- and TRF2-QDs, respectively (combined 125 and 225 mM ionic strength data). This data fitting scheme also provides confined DNA lengths of 0.61 (±0.09) and 0.4 (±0.09) μm, for TRF2 and TRF1, respectively.
The lifetime of protein attachment on DNA was determined by the length of the streak in the kymographic analysis. Only streaks that began and ended in a movie (Type I, Figure 2D) were analyzed, which may lead to a small systematic underestimation of the attached lifetime. The lifetime data were plotted as cumulative frequency (CF) histograms and fitted to:

$$CF = N(1 - e^{-k.t})/(1 - e^{-k.t_{max}})$$ **Equation 5**

where *N* is the number of observed points, *t* the bin, t_{max} the maximum bin size and *k* the reciprocal of the dwell time.

Due to the brightness variation of QDs, we were not be able to directly correlate the QD brightness and number of protein molecules on DNA.

Prediction of diffusion constants and stepping rates based on Stokes-Einstein relation

The hydrodynamic radii of red (655 nm) and green Sav-QDs (565 nm) are assumed to be 11.5 and 9.5 nm, respectively, based on recent measurements (46). The estimated radii of free TRF1 and TRF2 are 10 nm based on the crystal structures of the Myb type and dimerization domains (12,67). The expected upper limit for diffusion constants for a single red QD labeled TRF1 or TRF2 sliding on DNA is 17.8 $\mu m^2/s$. This is 187- to 468-fold higher than the measured diffusion constants for TRF proteins (between 3.8 $\times 10^{-2}$ and 9.5 $\times 10^{-2}$ $\mu m^2/s$, Table 1).
Assuming protein rotating around DNA helix, the expected upper limits for diffusion constants are based on the modified version of the Stokes-Einstein relation (68):

$$D_{1,cal} = \frac{K_B\, T}{6\pi\eta a\left[1+\left(\frac{4}{3}\right)(2\pi)^2\left(\frac{a}{3.4}\times 10^{-9}\right)^2\right]}$$ **Equation 6**

where η is viscosity of the medium, a the radius of the particle, K_B the Boltzmann constant, T the temperature.
The stepping rate can be calculated by assuming the diffusion constant to occur as a series of steps of a single base pair using the following relationship (69):

$$k = 2D(l_{bp})^2$$ **Equation 7**

where k is the stepping rate: Steps/s.

The expected upper limits for diffusion constants and stepping rates for TRF proteins (with one red QD) with rotation-coupled diffusion are 0.021 μm^2/s and 365492 steps/s, respectively. These numbers correspond to a diffusion rate of 0.42 μm^2/s and a stepping rate of 7315292 steps/s without QDs.

Prediction of additional energy barriers at telomeric sequences

The estimated minimal roughness of the energy landscape at specific binding sites is (35):

$\sigma = k_B T \sqrt{2 \ln M} = 6.6\, k_B T$ **Equation 8**

for a genome size of M = 3 x 10^9 bp.

The activation energy barriers (E_A) to protein diffusion can be calculated from stepping rates using Arrhenius relationship:

$k = e^{-E_A} / k_B T$ **Equation 9**

$E_A = -\ln(k) \cdot k_B T$

where k is the stepping rate: Steps/s.

Then the additional energy barrier at the telomeric region compared with the non-telomeric region can be calculated as:

$E_{A,tel} - E_{A,nontel} = -\ln(\frac{k_{tel}}{k_{nontel}}) \cdot k_B T$ **Equation 10**

In addition, the relative free binding energy at the telomeric regions with respect to non-telomeric regions can be defined as:

$\Delta\Delta G_{bind} = k_B T ln\left(\frac{K_{tel}}{Knontel}\right)$ **Equation 11**

where K_{tel} and K_{nontel} are equilibrium association constants at the telomeric and non-telomeric regions, respectively.

Assuming the association constants are the same at the telomeric and non-telomeric regions, Equation 11 can be expressed in terms of lifetimes of a protein on DNA (τ):

$\Delta\Delta G_{bind} = k_B T ln\left(\frac{\tau_{tel}}{\tau_{nontel}}\right)$ **Equation 12**

where τ_{tel} and τ_{nontel} are the lifetimes of a protein at the telomeric and non-telomeric regions, respectively.

Computer simulations of 1-D diffusion

Computer simulations of 1-D diffusion of proteins were carried out using Python^{TM} programming language 3.3.0. (Supplementary Figure S9). Nonspecific λ DNA was represented by a 1-D lattice without confinement. Since TRF2 and TRF1 have higher affinity for the telomeric sequences, we used 1.6 kb DNA with totally reflecting diffusion barriers at two ends to simulate TRF2 over the 1.6 kb $(TTAGGG)_{270}$ telomeric sequence flanked by non-telomeric sequences. In our simulations, we treated the telomeric region as a transient confinement zone, a concept developed for studying lipid rafts (70). The 1-D random walker starts at random locations inside the confined zone. To approximate

the positional averaging from the camera integration time required for imaging, the simulated position was averaged for each 50 ms period.

Accuracy of determining diffusion constant (*D*) and confinement length (*L*) values with camera time averaging

Camera time averaging has been a concern when trying to obtain diffusion constants for confined diffusion because a particle may diffuse a significant fraction of the corral size in time between consecutive measurements (71). However, previous theoretical modeling revealed that when τ, the dwell time to explore a 1-D lattice with a length of *L*, is large compared to the exposure time ΔT (50 ms), then the estimation of τ, *L* (confinement length), and *D* (diffusion constant) remain accurate (72). The accuracy of measuring diffusion constants from single-molecule experiment remains accurate for confinements with dwell times down to $\tau = \Delta T/3$ (72). The dwell time to explore a linear lattice with a confinement length of *L* is $\tau = L^2/(\pi^2 D)$ (73). In the case of TRF1 and TRF2 over the telomeric region, τ would be 10.0 s for a case with a diffusion constant of 0.3×10^{-2} um^2/s and *L* of 0.55 µm, corresponding to $\tau = 200\Delta T > \Delta T/3$. These parameters support the assumption that the measurement of diffusion constants in this study remains accurate even with camera time averaging.

Supplementary Movie Legends

1. **Movie S1. TRF1-QD on λ DNA.** A movie demonstrating real-time observation of 1-D sliding of a TRF1-QD on λ DNA at 75 mM ionic strength (real time, 34 s). The scale bar is 1 μm.
2. **Movie S2. TRF2-QD on λ DNA.** A movie demonstrating real-time observation of 1-D sliding of a TRF2-QD on λ DNA at 75 mM ionic strength (real time, 36 s). The scale bar is 1 μm.
3. **Movie S3. TRF1-QDs on the ligated T270 DNA.** Dual-color labeled TRF1-QDs on the ligated T270 DNA at 125 mM ionic strength (real time, 1 min 26 s). The scale bar is 1 μm.
4. **Movie S4. TRF2-QDs on the ligated T270 DNA**. Dual-color labeled TRF2-QDs on the ligated T270 DNA at 125 mM ionic strength (real time, 1 min and 16 s). The scale bar is 1 μm.
5. **Movie S5. TRF2-QDs on the ligated Tel10 DNA.** Dual-color labeled TRF2-QDs on the ligated Tel10 DNA at 125 mM ionic strength (real time, 49 s). The scale bar is 1 μm.

SUPPLEMENTARY REFERENCES

65. Takai, K.K., Hooper, S., Blackwood, S., Gandhi, R. and de Lange, T. (2010) In vivo stoichiometry of shelterin components. *The Journal of biological chemistry*, 285, 1457-1467.
66. Thompson, R.E., Larson, D.R. and Webb, W.W. (2002) Precise nanometer localization analysis for individual fluorescent probes. *Biophysical journal*, 82, 2775-2783.
67. Fairall, L., Chapman, L., Moss, H., de Lange, T. and Rhodes, D. (2001) Structure of the TRFH dimerization domain of the human telomeric proteins TRF1 and TRF2. *Molecular cell*, 8, 351-361.
68. Schurr, J.M. (1979) The one-dimensional diffusion coefficient of proteins absorbed on DNA. Hydrodynamic considerations. *Biophysical chemistry*, 9, 413-414.
69. Hughes, B.D. (1995) Random Walks. *Oxford University Press*, 1.
70. Dietrich, C., Yang, B., Fujiwara, T., Kusumi, A. and Jacobson, K. (2002) Relationship of lipid rafts to transient confinement zones detected by single particle tracking. *Biophysical journal*, 82, 274-284.
71. Saxton, M.J. (1995) Single-particle tracking: effects of corrals. *Biophysical journal*, 69, 389-398.
72. Destainville, N. and Salome, L. (2006) Quantification and correction of systematic errors due to detector time-averaging in single-molecule tracking experiments. *Biophysical journal*, 90, L17-19.
73. Meilhac, N., Le Guyader, L., Salome, L. and Destainville, N. (2006) Detection of confinement and jumps in single-molecule membrane trajectories. *Phys Rev E Stat Nonlin Soft Matter Phys*, 73, 011915.

Table S1

Percentages of four types of protein-DNA interactions for TRF1 and TRF2 on λ DNA and the ligated T270 DNA substrate at different ionic strengths.

Ionic Strength (mM)	DNA	TRF1-QDs (%)					TRF2-QDs (%)				
		n	I	II	III	IV	n	I	II	III	IV
75	λ DNA	179	5	2.2	16.8	76	278	37.4	8.3	16.5	37.8
125	λ DNA	63	7.9	1.6	11.1	79.4	171	62	4.1	15.2	18.7
175	λ DNA	210	30	4.8	10.5	54.7	177	60.4	3.4	13.6	22.6
225	λ DNA	291	44.3	1.7	6.2	47.8	154	61.7	5.2	11.7	21.4
125	T270	184	10.9	6	17.9	65.2	374	27	5.1	16.6	51.3
225	T270	110	4.5	5.5	27.3	62.7	216	43.5	3.2	24.1	29.2

Note: The examples of each type of kymograph are shown in Fig. 2*D*. The total video length was 2 minutes for λ DNA and 4 mins for the ligated T270 DNA substrate. Increasing the video length to 4 mins for λ DNA does not change the lifetime measurement for the Type I complexes.

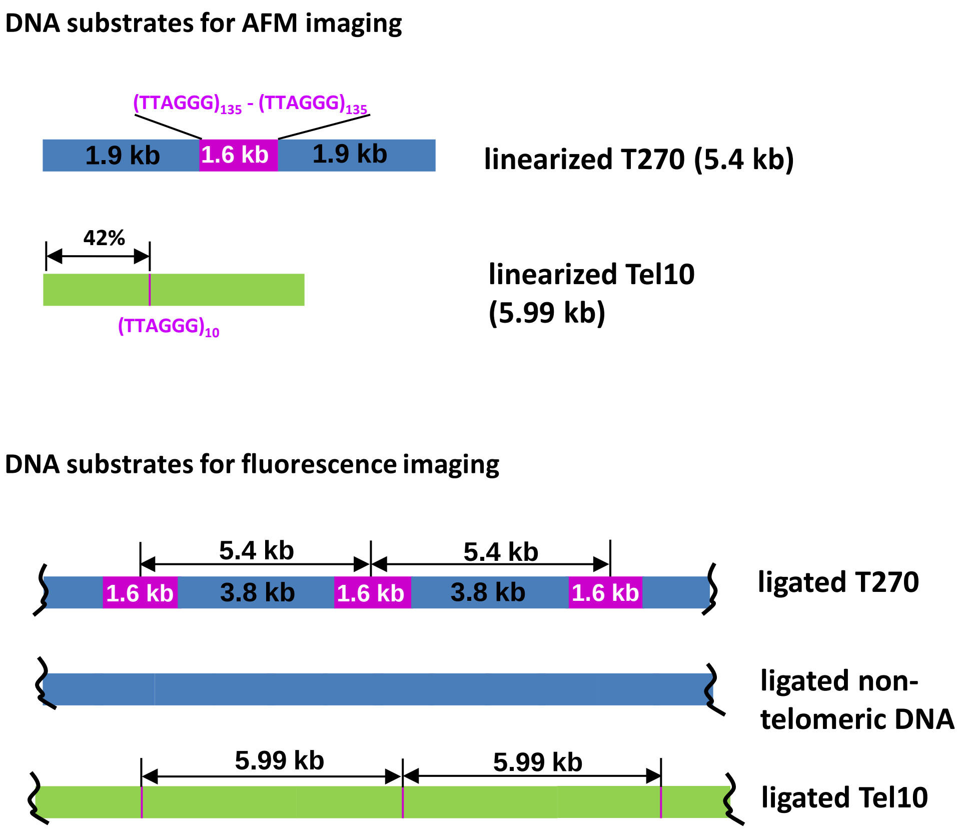

Figure S1. DNA substrates used for AFM and fluorescence imaging (not including λ DNA). The purple and blue/green regions represent telomeric and non-telomeric sequences, respectively. Ligated DNA substrates have heterogeneous lengths.

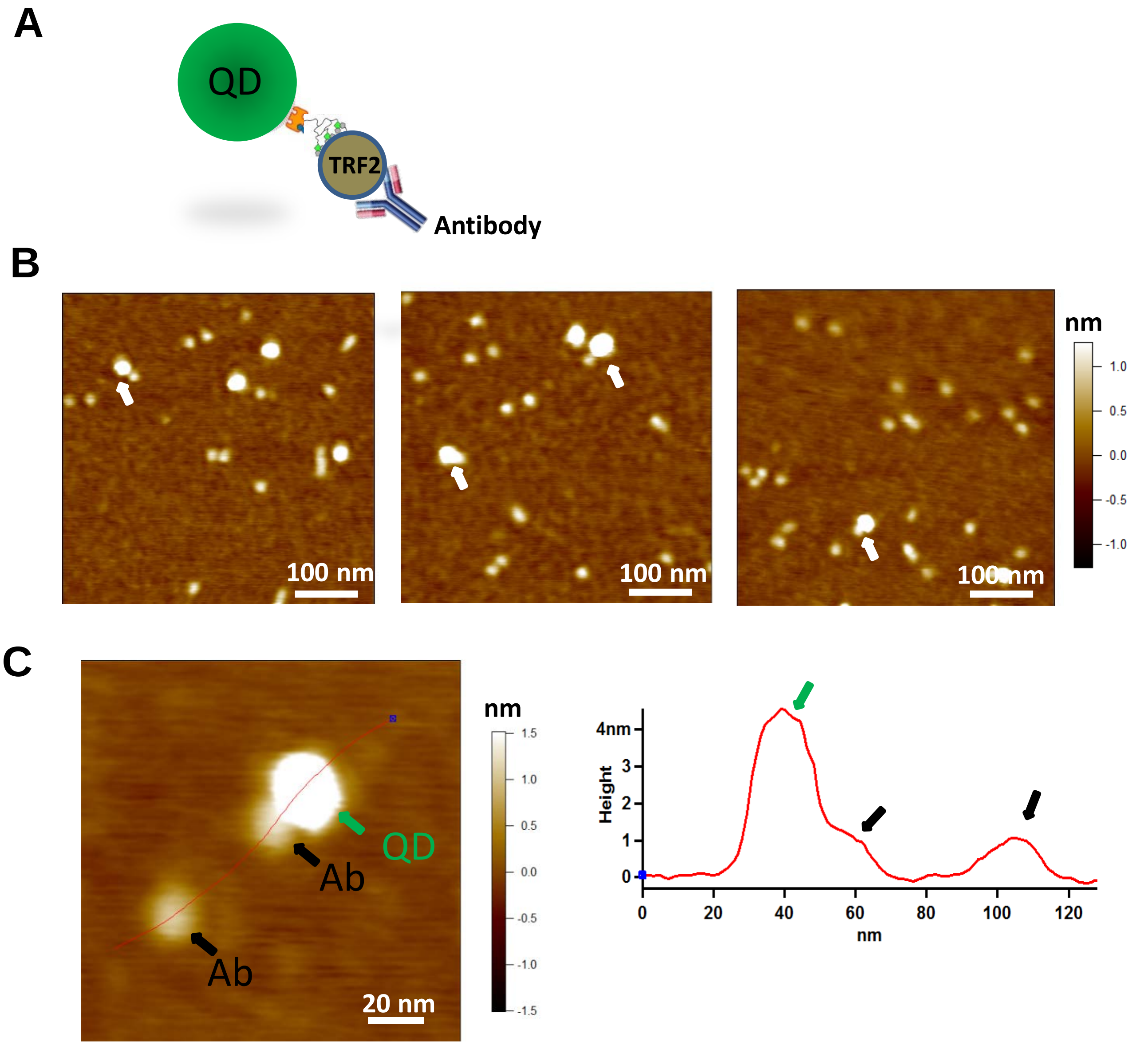

Figure S2. Stoichiometry of TRF2-QDs revealed by AFM imaging. (*A*) A schematic representation of TRF2 primary antibody binding to a TRF2-QD. (*B*) AFM images of TRF2-QDs in the presence of TRF2 primary antibody (TRF2:Ab = 1:1). White arrows point to TRF2-QDs with a single antibody. (*C*) Cross section analysis of a TRF2-QD complex binding to TRF2 antibody. The section analysis on the right is from the path drawn in the AFM image on the left (red line).

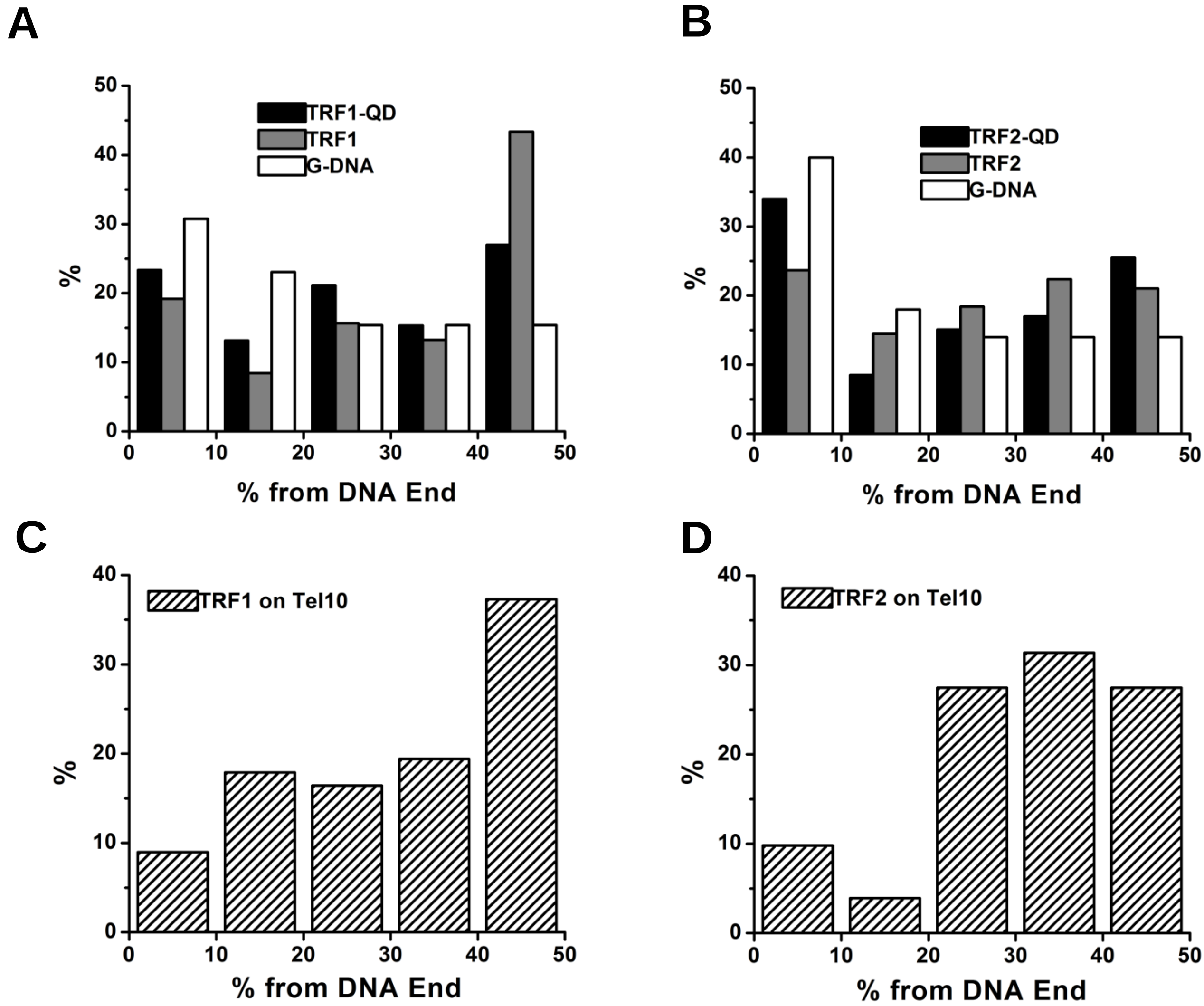

Figure S3. TRF1- and TRF2-QDs bind specifically to the telomeric sequences. (*A*) Position distributions of TRF1-QDs (black bars, n = 103) and unlabeled TRF1 (gray bars, n = 83) on the T270 telomeric substrate, and TRF1-QDs on the non-telomeric DNA substrate (white bars, n = 13). (*B*) Position distributions of TRF2-QDs (black bars, n = 106), unlabeled TRF2 (gray bars, n = 76) on the T270 telomeric substrate, and TRF2-QDs on the non-telomeric DNA substrate (white bars, n = 100). The $(TTAGGG)_{270}$ sequence makes up approximately 30% of the total DNA length and is located in the middle of the linearized T270 DNA (between 35% to 50% from one end). Among the protein-QDs on DNA, ~40.6% TRF1-QD and ~52.6% of TRF2-QD bound to the telomeric regions (excluding complexes bound right at the end of the DNA). The small peak at 24% is consistent with the locations of previously discovered preferred TRF1 and TRF2 binding sequence (CCATTC) over the non-telomeric region. (*C* and *D*) Position distributions of TRF1-QDs (*C*, n = 67) and TRF2-QDs (*D, n=51*) on linear Tel10 DNA substrate with $(TTAGGG)_{10}$ sequence located at 42% from one DNA end.

A

Percentages of motile TRF1- and TRF2-QDs on λ DNA.

Ionic Strength (mM)	TRF1-QDs n	TRF1-QDs %	TRF2-QDs n	TRF2-QDs %
75	84	27	166	97
125	87	33	142	95
175	116	30	154	95
225	135	15	158	89

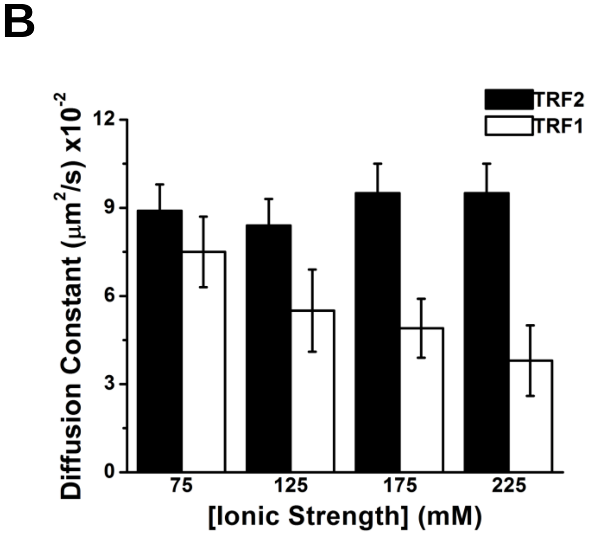

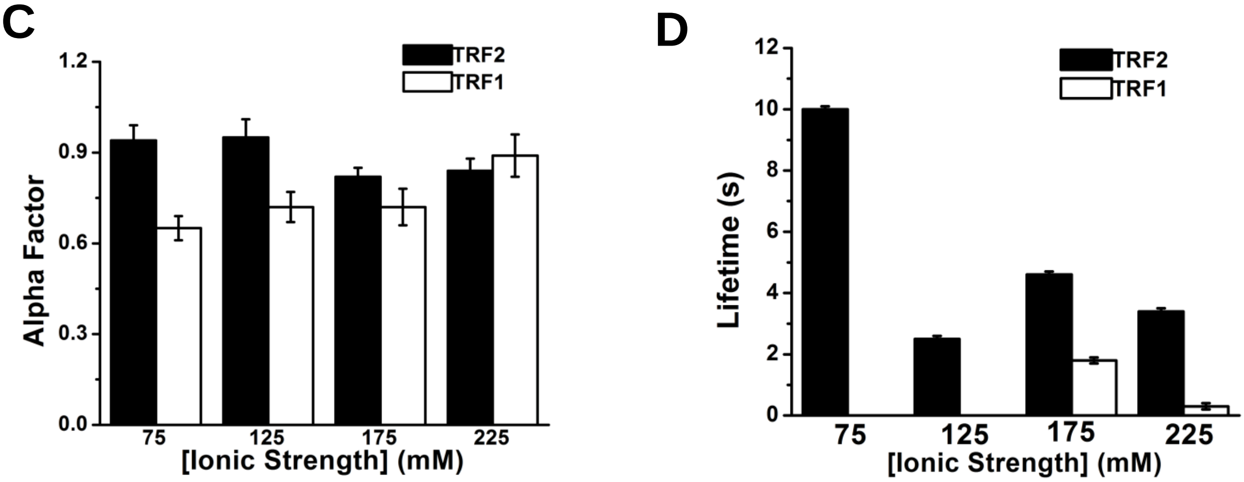

Figure S4. Diffusional properties of TRF1 and TRF2 on λ DNA. (*A*) Percentages of motile TRF1- and TRF2-QDs on λ DNA. n is the number of complexes analyzed. TRF proteins were labeled with red QDs (655 nm). (*B*) Diffusion constants of TRF1- (white bars) and TRF2-QDs (black bars) at different ionic strengths. (*C*) Alpha factors of TRF1- (white bars) and TRF2-QDs (black bars) at different ionic strengths. The numbers of the complexes analyzed were 51, 37, 40, and 33 for TRF1-QDs, and 59, 54, 63, and 66 for TRF2-QDs, at 75, 125, 175, 225 mM ionic strengths, respectively. (*D*) The effect of ionic strength on the lifetimes of TRF1- (white bars) and TRF2-QDs (Black bars) on λ DNA. The numbers of the complexes analyzed were 63 and 128 for TRF1-QDs, and 104, 106, 107, and 95 for TRF2-QDs with increasing ionic strengths. Across all ionic strengths, compared to TRF2, TRF1 showed lower affinity for nonspecific λ DNA, indicated by lower average numbers of protein-QDs (1.1 ± 0.1 vs. 13.3 ± 2.2) in each field of view (27 μm x 55 μm) and lower average numbers of TRF1-QDs on DNA tightrope(s) between two beads (1.1 ± 0.1 vs. 3.8 ± 0.3).

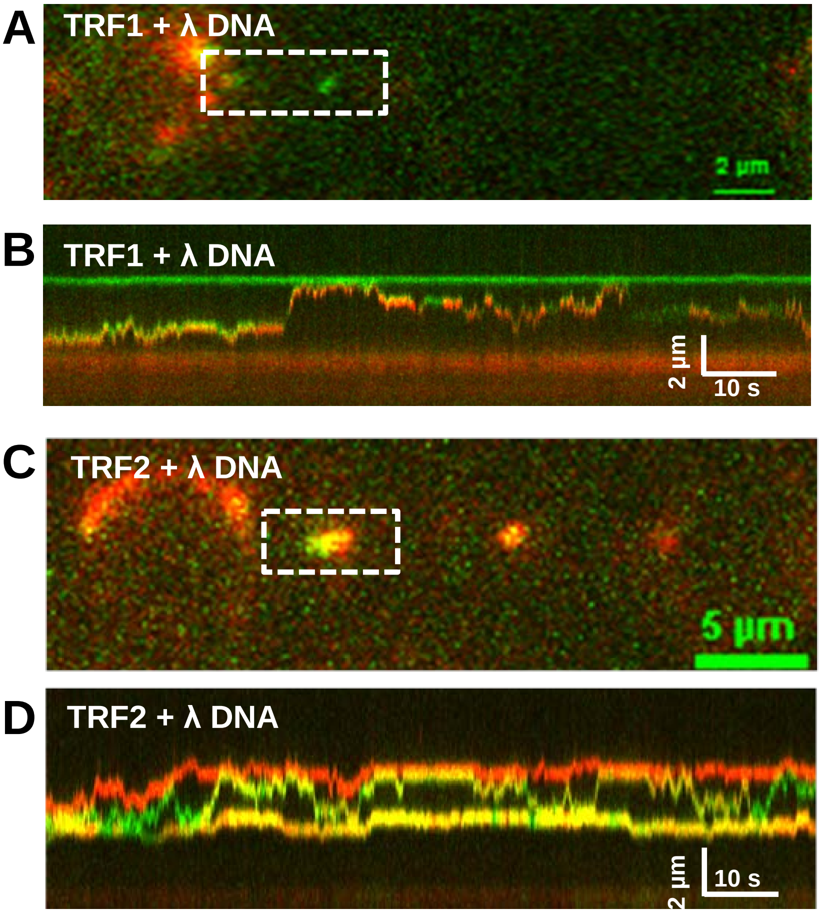

E

Percentages of dual color labeled TRF1 and TRF2 on λ DNA.

Complexes	TRF1-QDs	TRF2-QDs
Total number	141	43
Dual color (%)	19%	79%
Red (%)	41%	10%
Green (%)	39%	11%

Figure S5. Dual color labeling of TRF1 and TRF2. A representative fluorescence image (*A*) and kymograph (*B*) of dual-color labeled TRF1-QDs on λ DNA. A representative ORF image (*C*) and kymograph (*D*) of dual-color labeled TRF2-QDs on λ DNA. TRF1 or TRF2 was incubated with equal molar of red (655 nm) and green (565 nm) QDs. The kymographs shown in (*B*) and (*D*) are from the boxed regions in (*A*) and (*C*), respectively. Among the observed TRF2-QD complexes encountering another protein on DNA (n = 26), we did not observe any protein barrier bypass events for TRF2. Due to the lower binding affinity of TRF1 on DNA with non-telomeric sequences, even though we did not observe bypass when TRF1-QD complexes encountered barriers posed by other proteins (n = 3 out of 87 binding events), the number of events was not sufficient to make any statistical comparisons. (*E*) Percentages of dual color QD labeled TRF1 and TRF2.

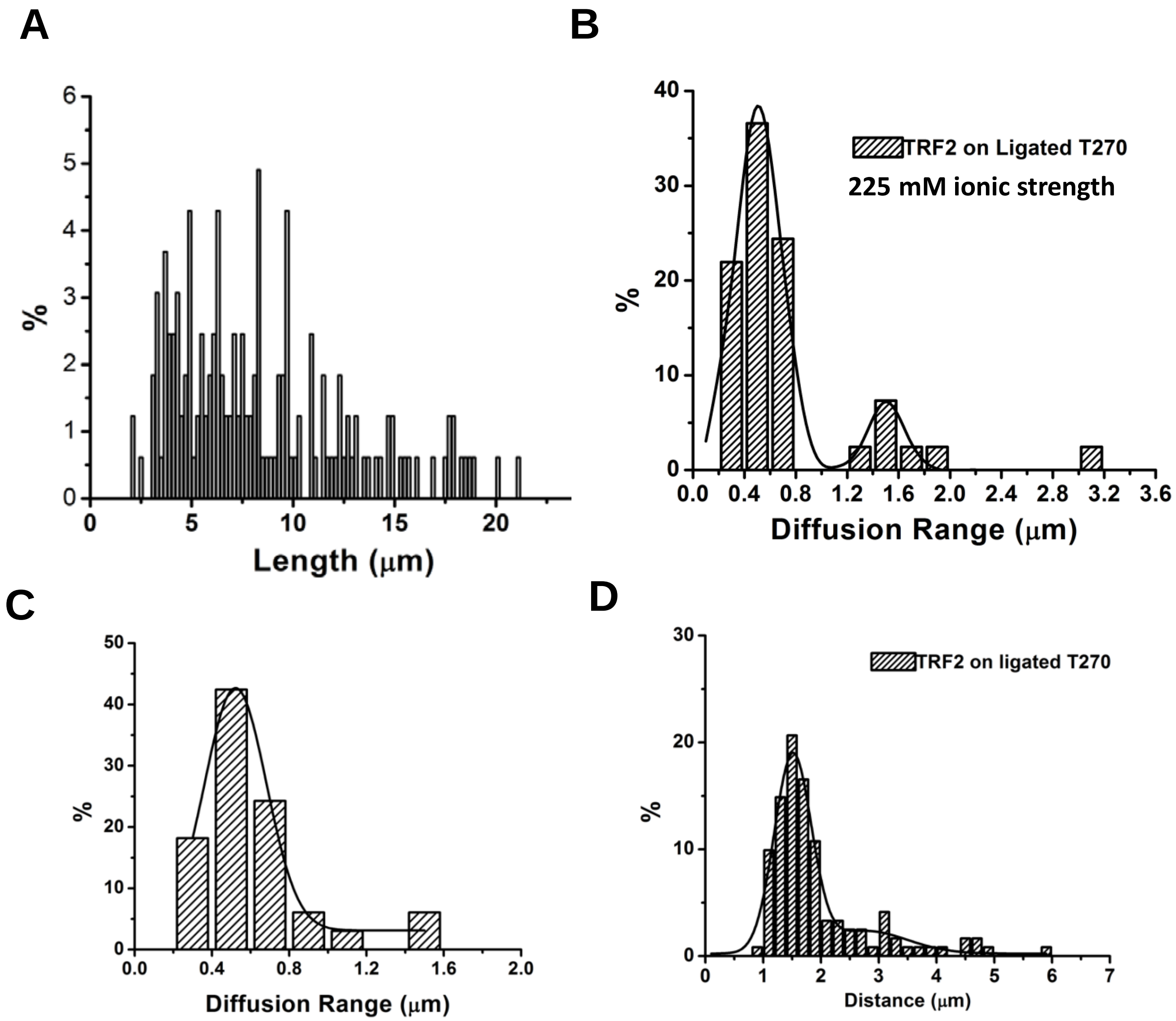

Figure S6. Additional information on the length of ligated T270 DNA tightropes, the diffusion range distributions, and distances between two adjacent TRF2-QDs on the ligated T270 substrate. (*A*) Measured ligated T270 DNA lengths between two anchoring silica beads (n = 163). (*B*) The diffusion range distribution of TRF2-QDs at 225 mM ionic strength (n = 4 1). The line in the plot is double Gaussian fit to the data with R^2 of 0.97 and peaks centered at 0.5 and 1.5 μm. (*C*) The diffusion range of TRF2-QDs on ligated T270 DNA at 0.68 nM TRF2 and 0.33 nM QD concentrations (n = 33). The line in the plot is single Gaussian fit to the data with R^2 of 0.97 and the peak centered at 0.5 μm. Individual DNA tightropes with one or two QDs (no contact during recording) were analyzed. (*D*) The distances between adjacent TRF2-QDs on DNA tightropes formed at 25 μl/ml flow rate (n = 121). The line in the plot is double Gaussian fit to the data with R^2 of 0.97 and peaks centered at 1.5 and 2.7 μm. The spacing between adjacent TRF2-QDs indicate that DNA was stretched to ~88% of its contour length.

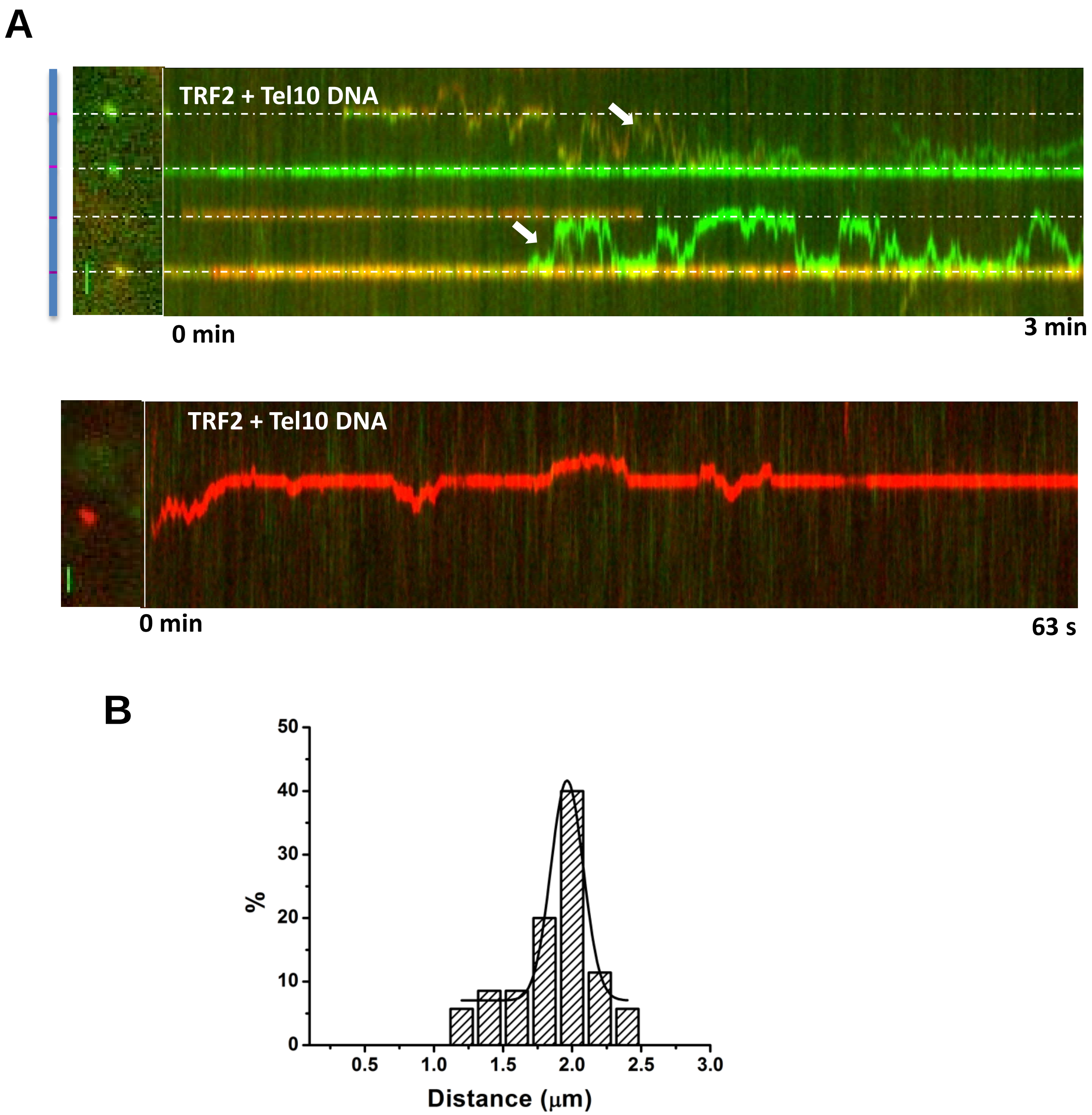

Figure S7. Dynamics of TRF2-QDs on the ligated Tel10 DNA. (*A*) Kymographs of TRF2-QDs on the ligated Tel10 DNA. On this substrate, there were two populations: static complexes with apparent diffusion constant of 2.5 (±0.05) $\times 10^{-4}$ $\mu m^2/s$ (n = 10) and complexes with long diffusion range (>850 nm, white arrows). (*B*) Measured distance between two adjacent TRF2-QDs on ligated T10 DNA substrate (n = 35). The line in (*B*) is Gaussian fit to the data, which has R^2 of 0.98. Consistent with a larger spacing (5.99 kb) between two adjacent telomeric regions on the Tel10 DNA substrate, static TRF2-QDs were spaced at longer distances (1.9 ± 0.049 μm) compared to that on the ligated T270 substrate (1.6 ± 0.01 μm, Fig. 3).

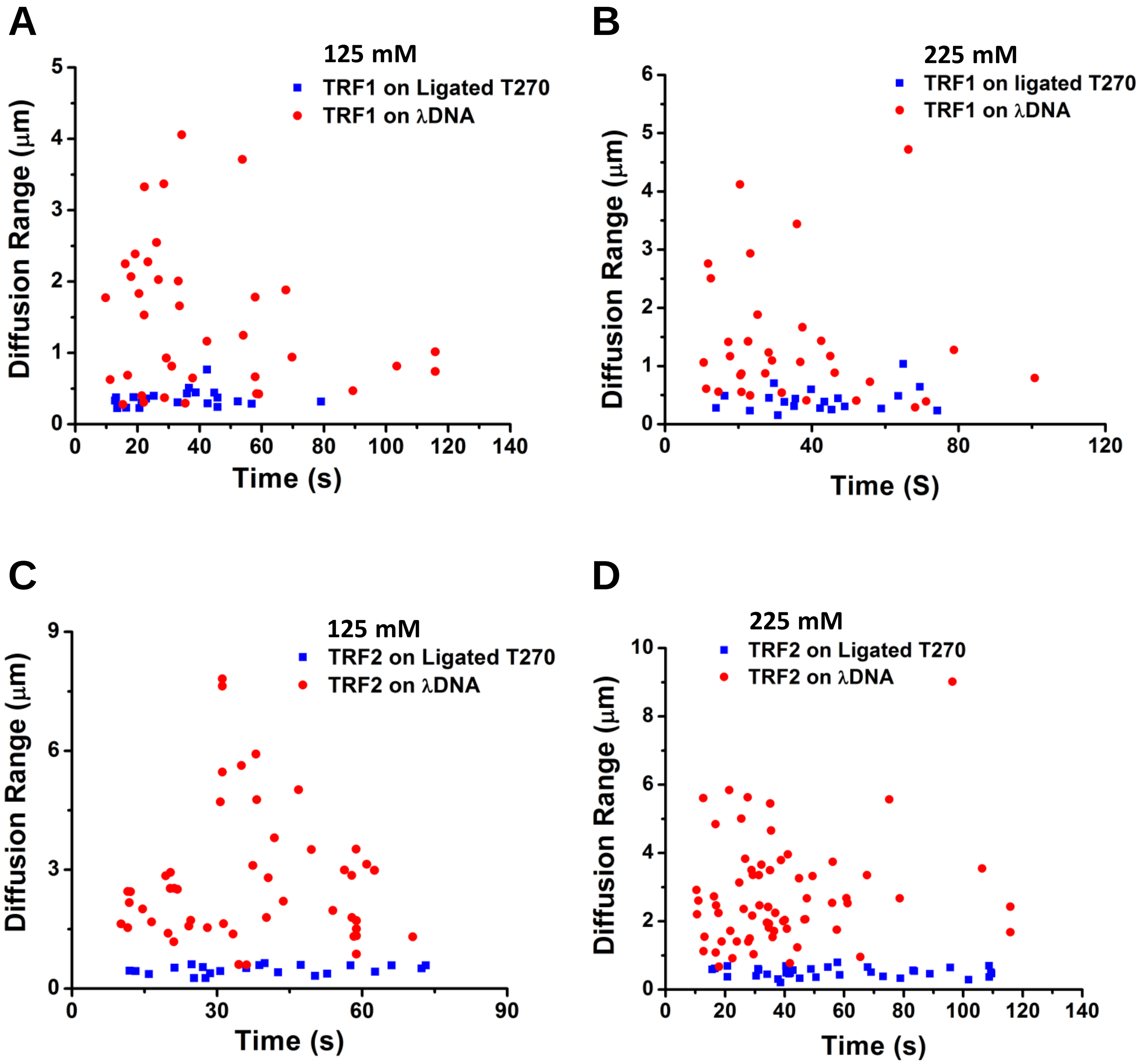

Figure S8. Short diffusion ranges of TRF1- and TRF2-QDs at $(TTAGGG)_{270}$ telomeric regions are narrowly distributed independent of the video length. The diffusion range over time for TRF1-QDs at 125 mM ionic strength (*A*) and 225 mM strength (*B*), and TRF2-QDs at 125 mM ionic strength (*C*), and 225 mM strength (*D*) on λ DNA (red dots) and the ligated T270 DNA (blue squares). The numbers of complexes plotted for TRF1 are 37 and 33 for λ DNA, 26 and 21 for T270 DNA, at 125 and 225 mM ionic strength, respectively. The numbers of complexes plotted for TRF2 are 48 and 66 for λ DNA, 22 and 34 for T270 DNA, at 125 and 225 mM ionic strength, respectively. On λ DNA, the diffusion ranges are widely distributed between approximately 0.5 to 9 μm. The time scale (from 10 to 120 s) is from the cropped videos used for the diffusion constant and diffusion range analysis.

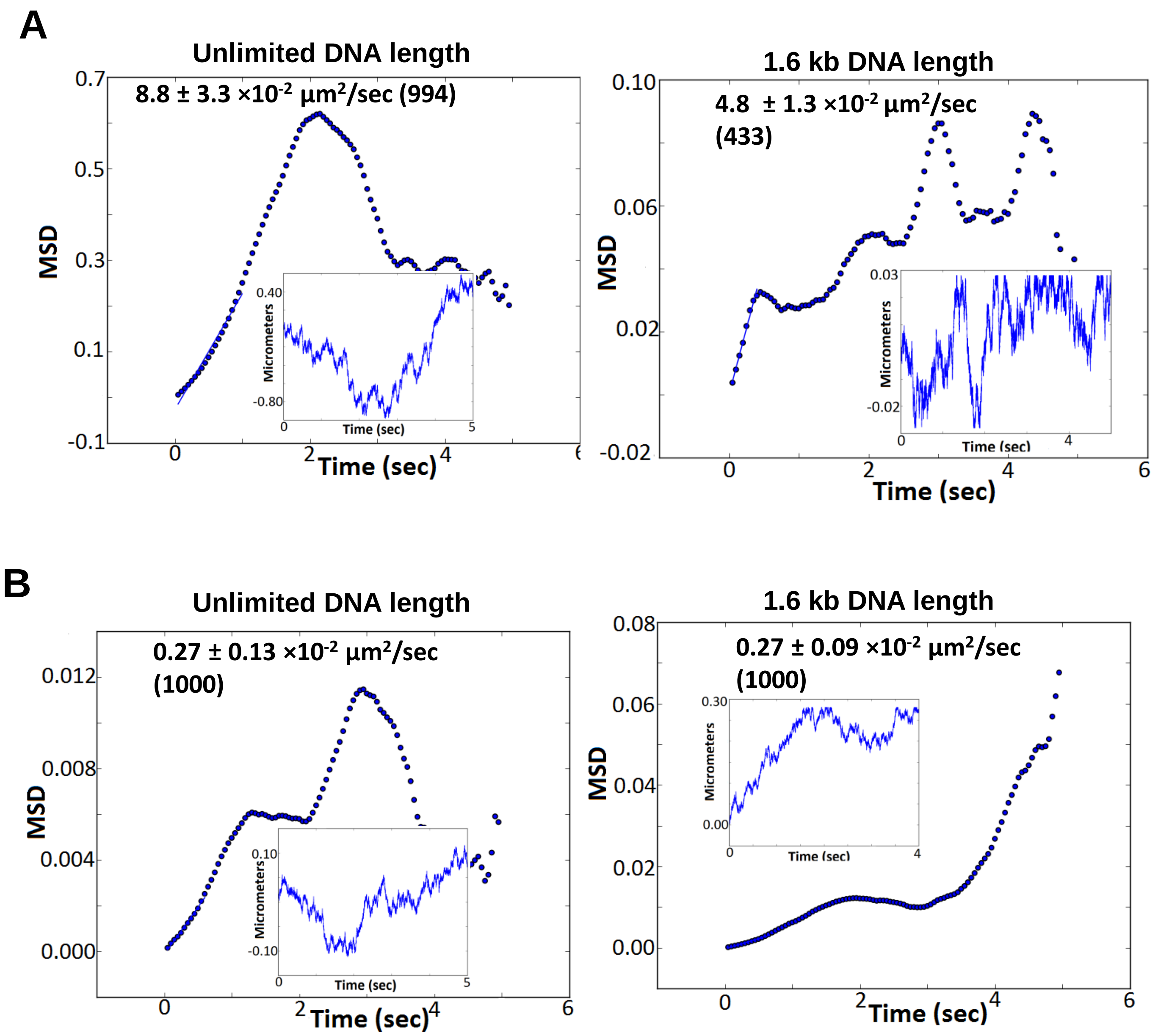

Figure S9. Computer simulations of diffusion by modeling random walk of proteins on a 1-D DNA lattice using Python™ programming language. The diffusion constants used for simulation were that of TRF2-QDs on λ DNA (*A*) and on the telomeric DNA (*B*) at 125 mM ionic strength. A plot for MSD vs. Δt obtained and an example of a trajectory of a protein (insert) are presented for (left, *A*) DNA with unlimited length and (right, *A*) DNA with 1.6 kb length at 1460436 steps/s stepping rate (corresponding to TRF2-QDs on λ DNA). A plot for MSD vs. Δt and an example of a trajectory of a protein (insert) are also presented for (left, *B*) DNA with unlimited length and (right, *B*) DNA with 1.6 kb length at 46713 steps/s (corresponding to the rate of TRF2-QDs at the telomeric region). The fitting parameters were constructed such that only the initial linear portion of the MSD vs. Δt plots was used for calculating the diffusion constant. For diffusion with confinement, a protein walks along a 1-DNA lattice with two totally reflecting barriers. The numbers in each plot are the mean and standard deviation of the simulated data. The number of particles simulated for each case is indicated in the parentheses.

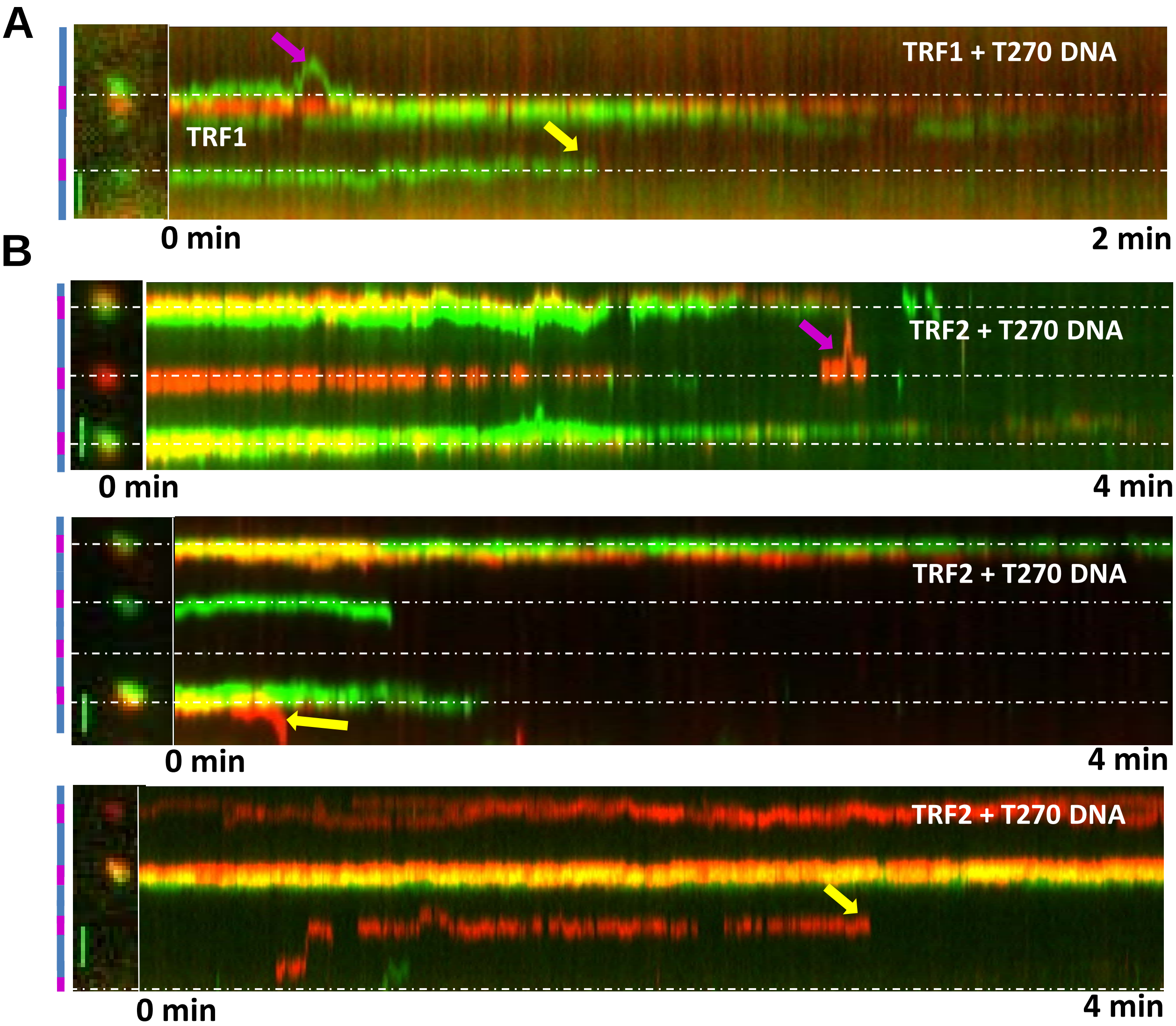

Figure S10. Dissociation events of TRF1 and TRF2-QDs on the ligated T270 DNA. Representative kymographs of TRF1- (*A*) and TRF2-QDs (*B*) on the ligated T270 DNA at 125 mM ionic strength. The scale bar is 1 µm. The yellow arrows point to dissociation events either directly from the telomeric region or through the non-telomeric region. The purple arrows point to the transient dissociation and rebinding at the same telomeric region.

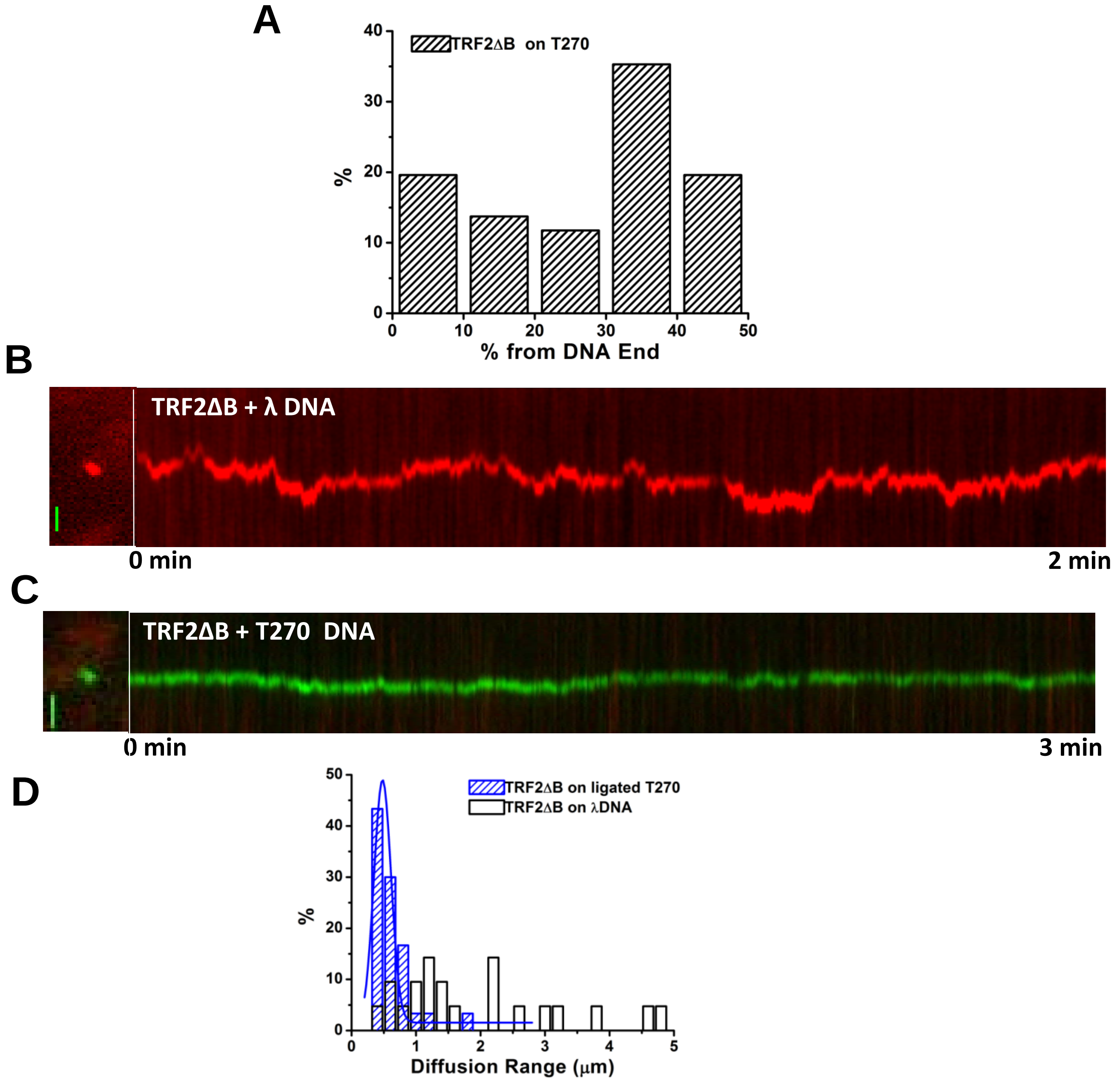

Figure S11. Dynamics of TRF2ΔB-QDs on λ DNA and the ligated T270 DNA. (*A*) Position distributions of TRF2ΔB-QDs on the linear T270 DNA substrate (n = 51). Among the protein-QDs on DNA, ~66% TRF2ΔB-QDs bound to the telomeric regions (35% to 50% from DNA ends). Kymographs of TRF2ΔB-QDs on λ DNA (*B*) and the ligated T270 DNA (*C*). The scale bar is 1 μm. Protein-QD-DNA reactions were carried out at 125 ionic strength. (*D*) Diffusion range distributions of TRF2ΔB-QDs on λ DNA (white bars, n = 21) and the ligated T270 DNA (stripped bars, n = 30). The binding affinity of TRF2ΔB to λ DNA tightropes was significantly lower as indicated by lower average numbers of TRF2ΔB-QDs on DNA in the field of view (1.4 ± 0.2 vs. 13.3 ± 2.2) and lower average numbers of protein-QDs on DNA tightropes between two beads (1.1 ± 0.1 vs. 3.8 ± 0.3). Dual color QD labeling confirmed that TRF2ΔB can form dimers or higher order oligomeric species, but at a significantly lower percent (14%) compared with the full length protein (79%). The fraction of motile protein-DNA complexes decreased from 95% for full length TRF2 to 65% (n = 40) for TRF2ΔB at 125 mM and from 89% to 74% (n = 23) at 225 mM ionic strength.
